# Burrows of the Semi-Terrestrial Crab *Ucides cordatus* Enhance CO_2_ Release in a North Brazilian Mangrove Forest

**DOI:** 10.1371/journal.pone.0109532

**Published:** 2014-10-14

**Authors:** Nathalie Pülmanns, Karen Diele, Ulf Mehlig, Inga Nordhaus

**Affiliations:** 1 Department of Ecology, Leibniz Center for Tropical Marine Ecology (ZMT), Bremen, Germany; 2 School of Life, Sport and Social Sciences, Edinburgh Napier University, Edinburgh, United Kingdom; 3 Instituto de Estudos Costeiros, Universidade Federal do Pará - Campus de Bragança, Bragança, PA, Brazil; Agharkar Research Institute, India

## Abstract

*Ucides cordatus* is an abundant mangrove crab in Brazil constructing burrows of up to 2 m depth. Sediment around burrows may oxidize during low tides. This increase in sediment-air contact area may enhance carbon degradation processes. We hypothesized that 1) the sediment CO_2_ efflux rate is greater with burrows than without and 2) the reduction potential in radial profiles in the sediment surrounding the burrows decreases gradually, until approximating non-bioturbated conditions. Sampling was conducted during the North Brazilian wet season at neap tides. CO_2_ efflux rates of inhabited burrows and plain sediment were measured with a CO_2_/H_2_O gas analyzer connected to a respiration chamber. Sediment redox potential, pH and temperature were measured in the sediment surrounding the burrows at horizontal distances of 2, 5, 8 and 15 cm at four sediment depths (1, 10, 30 and 50 cm) and rH values were calculated. Sediment cores (50 cm length) were taken to measure the same parameters for plain sediment. CO_2_ efflux rates of plain sediment and individual crab burrows with entrance diameters of 7 cm were 0.7–1.3 µmol m^−2 ^s^−1^ and 0.2–0.4 µmol burrows^−1 ^s^−1^, respectively. CO_2_ released from a *Rhizophora mangle* dominated forest with an average of 1.7 *U. cordatus* burrows^−1 ^m^−2^ yielded 1.0–1.7 µmol m^−2 ^s^−1^, depending on the month and burrow entrance diameter. Laboratory experiments revealed that 20–60% of the CO_2_ released by burrows originated from crab respiration. Temporal changes in the reduction potential in the sediment surrounding the burrows did not influence the CO_2_ release from burrows. More oxidized conditions of plain sediment over time may explain the increase in CO_2_ release until the end of the wet season. CO_2_ released by *U. cordatus* and their burrows may be a significant pathway of CO_2_ export from mangrove sediments and should be considered in mangrove carbon budget estimates.

## Introduction

In mangrove ecosystems, semi-terrestrial burrowing crabs are important bioturbators affecting the biogeochemistry of the sediment and carbon cycling [1]–[5]. Their bioturbation activity includes constructing and maintaining of burrows leading to the reworking of sediment particles and organic material. Organic material, such as litter, is also reworked by their feeding, ingestion and defecation and can become buried in the sediment leading to carbon storage [1], [6]–[8]. Crabs accumulate carbon as body biomass by feeding on mangrove litter which otherwise would have been washed away by the tide [9]–[14]. However, crabs may also account for carbon depletion in the sediment. Their burrows present an extension of the sediment surface area [1], [15], thus, at low tide sediment surrounding the burrows can become oxidized by atmospheric oxygen. Atmospheric oxygen enters only a few millimeters into the mangrove sediment [8], [16], [17] where oxic reduction can take place. During burrow maintenance, crabs may mix more oxidized sediment inside their burrows with surrounding less oxidized sediment. The resulting increase in reduction potential affects the sediment surrounding the burrows up to a distance of several centimeters [18], [19]. This may alter the type of microbial carbon oxidation from mainly anaerobic (e.g. sulfate reduction or methanogenesis) to partially aerobic decomposition processes (e.g. aerobic respiration or iron reduction) [1], [8], [20], [21], which can result in higher CO_2_ efflux rates [22] and consequently to carbon loss from the sediment [6]. Thus, bioturbation by crabs and associated changes in the microbial decomposition process possibly influence CO_2_ release from the sediment.

To better evaluate the role of burrowing crabs in carbon cycling, it is important to quantify the amount of carbon stored and released by the crabs and their burrows. Bouillon et al. [23] assume that the CO_2_ released by burrows, animal respiration and roots is currently underestimated and may be an uncertain factor within the global carbon budget estimates for mangrove forests. Studies investigating the CO_2_ release by crab burrows or the reduction potential in the sediment surrounding the burrows have only been conducted for the relatively small fiddler crabs [18], [19], [24]. Inhabited fiddler crab burrows increased the CO_2_ release from mangrove sediment approximately 2 to 5 times compared to plain sediment [19], [24]. 58% of the released CO_2_ originated from the sediment surrounding the burrows and the remainder from the crab inside the burrow [24]. Studies characterizing the reduction potential in sediment surrounding fiddler crab burrows in salt marsh and mangrove sediments found a 1.5–4 cm thick oxidized zone, where iron reduction replaced the usually predominant sulfate reduction [18], [19]. This zone also presented the highest CO_2_ production rates. The rates decreased with increasing distance from the burrow, indicating that the change in reduction potential can influence carbon decomposition processes [18]. The availability and quality of carbon as well as the temperature can further influence carbon decomposition processes and thereby CO_2_ efflux from the sediments [8], [21].

Araújo Jr. et al. [25] investigated the bioturbation activity of the large and abundant crab *Ucides cordatus* (Ucididae), which, together with much smaller fiddler crabs, influence with their burrows the microtopography of the sediment in Brazilian mangrove forests. Araújo Jr. et al. [25] concluded from measurements of the reduction potential in sediment cores from bioturbated and non-bioturbated sites that *U. cordatus* burrows oxidized the sediment. However, the study did not provide information on the supposed gradient and the horizontal range of the reduction potential in the sediment surrounding the burrows, likewise, no CO_2_ efflux rates were measured.

In the present study the effects of bioturbation by *U. cordatus* on carbon dynamics and sediment reduction potentials were evaluated by (i) quantifying the CO_2_ efflux rate of individual burrows compared to plain sediment and (ii) measuring the reduction potential in radial profiles in the sediment around burrows at different depths in a *Rhizophora mangle* forest, respectively. We hypothesized that (i) sediment with *U. cordatus* burrows has higher CO_2_ efflux rates than sediment without burrows and (ii) reduction potentials in radial profiles of the sediment surrounding the burrows down to 50 cm depth decrease gradually from the burrow wall, approximating conditions of non-bioturbated sediment.

## Materials and Methods

The artisanally harvested study species *Ucides cordatus* is listed as endangered in the Brazilian legislation due to the risk of overfishing. All research activities were allowed by a research permit granted by SISBIO (Sistema de Autorização e Informação em Biodiversidade, authorization number 30007-1). Field work was performed in the extractive reserve “Reserva Extrativista Marinha de Caeté-Taperaçu”.

### Study area

**Figure 1:**
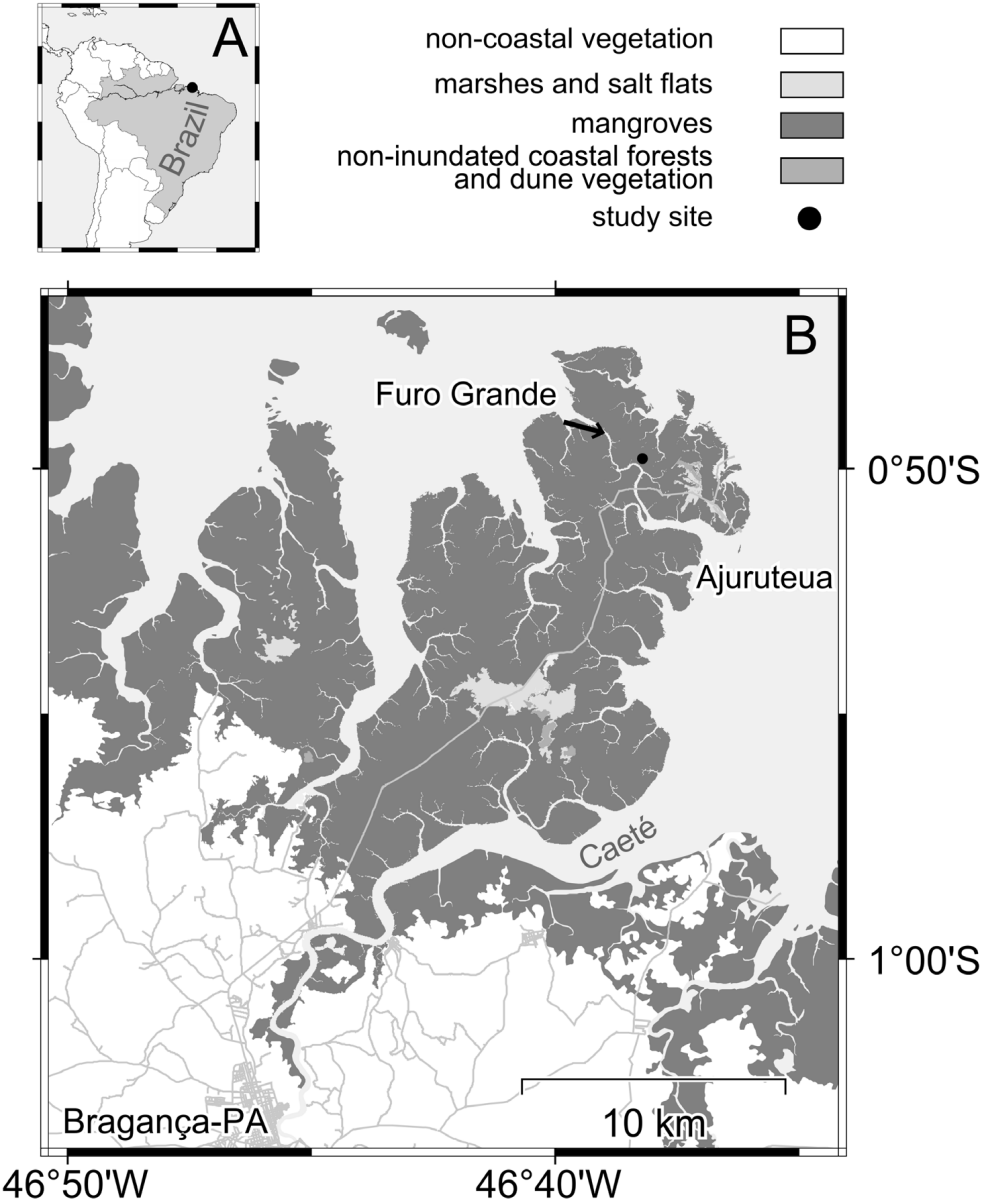
Study area on the Ajuruteua peninsula, Northern Brazil. The study was performed in Brazil (A) near the city of Bragança (46°38′W 0°50′S, B). The study site is marked by a black dot and is located at the tidal channel Furo Grande (B).

The study area is located on the Ajuruteua peninsula, the western bank of the Caeté river estuary in North Brazil (Figure 1). Field work was performed in the high intertidal zone near the tidal channel Furo Grande (46°38′W 0°50′S) (Figure 1, B). The region has semidiurnal tides with amplitudes of 2 to 5 m. During spring tide days the study site is flooded twice a day, while during neap tide days tidal heights are too low to inundate the whole mangrove forest [26], [27]. The forest is dominated by the mangrove tree *Rhizophora mangle* (Rhizophoraceae). Other mangrove tree species are also present including *Avicennia germinans* (Acanthaceae) and *Laguncularia racemosa* (Combretaceae) [26].

**Figure 2:**
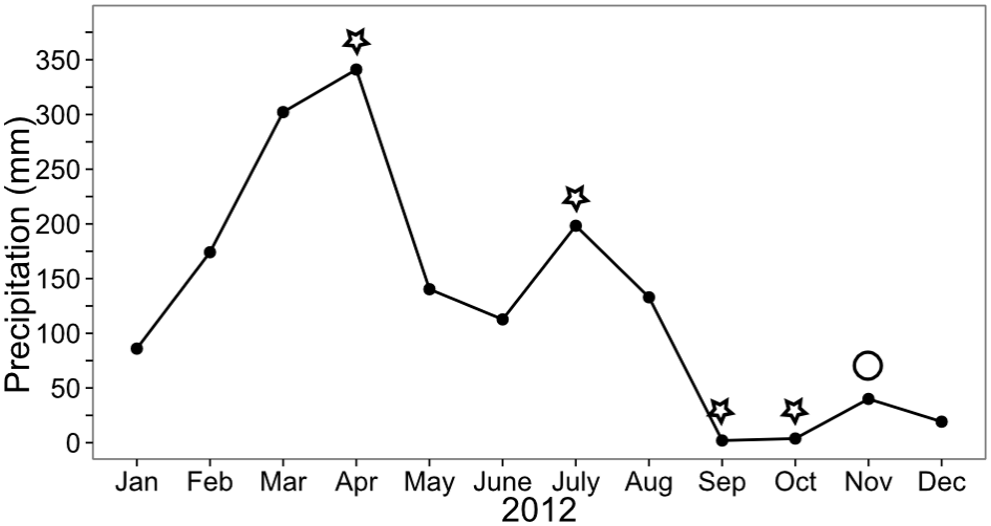
Total monthly precipitation in 2012. Precipitation (mm) was recorded by the weather station in Tracuateua (50 km southwest from the study site), Pará, Brazil [28]. The asterisks mark the sampling months (CO_2_ efflux rate and rH of burrows and control sediment). The circle marks the only control sampling (CO_2_ efflux rate and rH), which had to be conducted two neap tide cycles later than the corresponding sampling in October 2012 due to technical reasons.

Mean annual temperature in 2012 was 26.3±1°C (mean ± standard deviation, [28]). Total precipitation recorded at the weather station in Tracuateua (50 km from the study area) was 1552 mm (Figure 2). In Northern Brazil, the wet season generally occurs from January to August and the dry season (monthly precipitation <100 mm) from September to December [28]. Surface water salinity of the tidal channel Furo Grande varied between 22 and 37 throughout the year 2012 (unpublished data).

**Figure 3:**
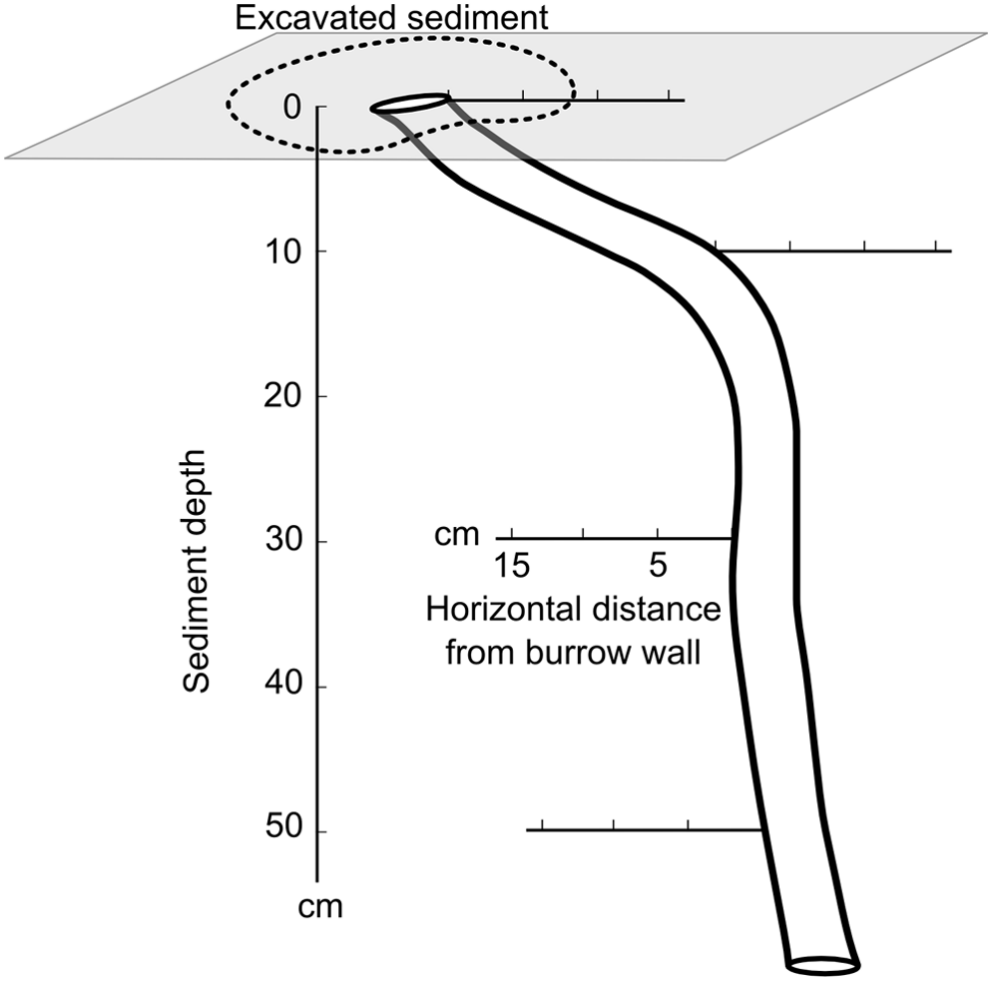
Vertical and horizontal sampling scheme of the sediment surrounding *U. cordatus* burrows for the rH measurements.

In the study area *U. cordatus* is the only large burrowing crab (max. carapace width of 9.9 cm in the Caeté estuary, [29]). An average density of 1.7 ind. m^−2^ was reported for the Furo Grande area in *R. mangle* dominated forests [30]. *U. cordatus* prefers to construct its burrow around the root system of *R. mangle*, because large roots structure the sediment and thereby increase sediment stability and provide shelter against predators [31]. Burrows of *U. cordatus* can have various forms. They can be simple burrows with one opening and one corridor, U-shaped with more than one opening, or even more complex structures with a maximum of three openings [32]. In northeast Brazil simple burrows are the most common type (more than 85% of all investigated burrows, n = 735) [32]. In the Furo Grande area simple burrows are also the most common burrow type (unpublished observation). Such burrows have the following morphology: Their corridors initially descend with a slight slope and then bend vertically downwards until forming a terminal burrow chamber ([14], Figure 3). At our study site at the Furo Grande total burrow corridor lengths (initial horizontal and vertical part) ranged from 73 to 219 cm with an average of 123±32 cm (mean ± standard deviation, n = 30, own data). The inner walls of the burrows of *U. cordatus* have no conspicuous inner mucus layer such as known from Polychaetes (e.g. reviewed by Kristensen and Kostka [33]). The burrows of the other crab species, for example sympatric fiddler crabs, also seem to be unlined (own observation, [34]).

### CO_2_ efflux rates

CO_2_ efflux rates of both burrows and plain sediment were measured in April, July, September and October 2012 around neap tides during the daytime. Due to technical reasons, measurements of sediment with burrows and plain sediment were performed during two succeeding neap tide cycles. In October 2012, the sampling of plain sediment was performed two neap tide cycles later.

*U. cordatus* burrows with only few *R. mangle* stilt roots nearby were chosen for the measurements to minimize the effect of root respiration. Furthermore, freshly excavated and blackish sediment had to be in front of their entrances indicating the presence of a live crab and ensuring sampling of recently bioturbated sediment. The diameter of the burrow entrances and if possible the current depths from the sediment surface to the water surface inside the burrow were recorded with a measuring tape before taking measurements. If the water surface inside the burrows was too low to measure, measurement was delayed to the rH measurements, when parts of the burrow walls were removed to facilitate access. The area of the burrow wall exposed to air was estimated based on the entrance diameter and the distance to the water surface.

For measuring the CO_2_ efflux rate of a burrow, a PVC collar with 20 cm diameter was positioned around the entrance and slightly pushed several centimeters into the sediment. To avoid any influence of CO_2_ released during and after the installation of the collar, the measurements were delayed for 1 h. A respiration chamber connected to a CO_2_/H_2_O infrared gas analyzer (LI-8100A, LI-COR, Biosciences) was then placed on top of the PVC collar. Since the respiration chamber was opaque no CO_2_ was consumed through photosynthesis during measurements. The CO_2_ efflux was recorded over 2 min and the measurement repeated five times. Between replicates, the chamber was opened for 25 s to release the accumulated CO_2_. Most probably, these measurements included the respiration of the crab inside the burrow. The contribution of crab respiration to the measured CO_2_ efflux rate was estimated by a separate experiment (see below). Sediment temperature was measured outside the collar at 2 cm sediment depth with a thermocouple (OMEGA Engineering).

As a control, the CO_2_ efflux rate of plain sediment without *U. cordatus* burrows and at the most with very few fiddler crab burrows was performed, following the same measurement protocol as for the sediment with burrows. However, four replicates were taken, because preliminary tests had shown that variance of measured values for plain sediment was smaller than for sediment with *U. cordatus* burrows.

The CO_2_ efflux rates were calculated for a linear flux rate [35] taking into account the surface sediment temperature and surface area inside the collar. For the measurement with *U. cordatus* burrows, the surface area inside the collar also included the burrow wall surface area. It also had to be considered that the relationship between burrow opening area and surface sediment area inside the collar did not represent the average relationship of burrow opening area to plain sediment area at the study site due to specific selection of single burrows and the small collar area. Under natural conditions, the proportion of burrow to plain area is lower (1.7 ind. m^−2^, [30]). Therefore, the calculated CO_2_ efflux rates inside collars with a crab burrow could not be directly compared with rates of plain sediment. To nevertheless allow for a comparison between the CO_2_ efflux rates of plain sediment with sediment containing burrows at a given burrow density, the following calculations were performed: First, the CO_2_ efflux rates of crab burrows without the surface sediment were estimated. To do this, the corresponding monthly mean CO_2_ efflux rates of the plain sediment measurements were subtracted from the calculated CO_2_ efflux rate of the burrows, considering the areal proportions of each term as follows:
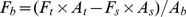



*F* is the CO_2_ efflux rate and *A* the area, which are both linked to plain sediment *s*, burrow *b*, total measured area *t* or total CO_2_ efflux *t*. The total measured area refers to the surface area inside the PVC collar. Mean control CO_2_ efflux rates of each month were used for *F_s_.* From this calculation values for *F_b_* (µmol m^−2 ^s^−1^) were obtained. From these values we derived CO_2_ efflux rate of individual burrows (µmol burrows^−1 ^s^−1^) with entrance diameters of 5, 6 and 7 cm corresponding to the range of burrow diameters sampled in the field. CO_2_ efflux rates for mangrove sediment based on the CO_2_ efflux rate of plain sediment and individual burrows were then obtained with the help of size-specific crab burrow density data. Two burrow densities were chosen, one represents the mean *U. cordatus* burrow density in a *R. mangle* dominated forests with 1.7 ind. m^−2^ for the Furo Grande area [30]. To simplify the calculation, this density of crabs can be related to 1.7 burrows^−1 ^m^−2^ since most *U. cordatus* burrows have only one opening. The other chosen burrow density was 11.9 burrows^−1 ^m^−2^ (unpublished data) representing the increased burrow density around dense *R. mangle* stilt roots.

### Respiration of *U. cordatus*

Measuring the CO_2_ efflux rate of a burrow without a live crab was not feasible as removal of the crab would have strongly disturbed the sediment. To nevertheless allow an estimation of the proportion of the CO_2_ efflux rate contributed by the crabs, their respiration rates were determined in an additional experiment. Adult male *U. cordatus* were caught nearby the study site. The carapace width (cm) of each animal was measured and the wet mass (g) was calculated by the regression formula for male *U. cordatus* provided by Diele [36]:
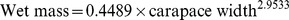



In the laboratory, crabs were placed in a PVC basin (20 cm diameter, 6 cm height) with the respiration chamber mounted on top. Under natural conditions crabs rest in air and water (own observation). Therefore, measurements were performed with different crabs in a dry basin (1. treatment: respiration in air, n = 14) and in a basin containing seawater (2. treatment: respiration in seawater, water level 4 cm, salinity 32, n = 14). CO_2_ released by each crab was measured five times for 10 min with breaks of 25 s in-between to open the chamber and release the accumulated CO_2_. Crabs were not acclimated inside the chamber prior to measurements. Therefore, the five consecutive measurements presented a gradient of crab respiration from a least acclimated state to a more acclimated one. CO_2_ respiration rates of crabs (µmol CO_2 _kg^−1 ^s^−1^) were calculated as described above, but related to wet mass instead of surface area in the calculation.

In the next step the contribution of crab respiration to the CO_2_ efflux rate was estimated for individual burrows with a burrow entrance diameter (BED) of 5, 6 and 7 cm. Since the inhabitants of the burrows used for CO_2_ measurements were not captured, their carapace width was estimated from the BED by a least squared regression formula based on data collected by Piou et al. [31]:
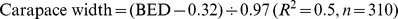



The wet mass was then estimated from the calculated carapace width with the formula for male *U. cordatus* by Diele [36] as indicated above. With the estimated wet mass of crabs inhabiting burrows with entrance diameters of 5, 6, and 7 cm, respectively, the respiration rate of individual crabs (µmol CO_2_ crab^−1 ^s^−1^, in air and in seawater) was calculated based on the first and the last measurement of the five consecutive respiration measurements. These two measurements represent a “worst” (crab least acclimated, resting in air) and a “best” case scenario (crab with the longest acclimatization time and resting in water), since preliminary tests had shown that crabs resting in air had higher CO_2_ efflux rates compared to crabs resting in water.

### Reduction potential

After the CO_2_ efflux rate measurements in the field were performed, reduction potentials of both burrow walls and of non-bioturbated sediment were measured at the selected sites on the same day. Redox potential (±1.0 mV), pH (±0.1) and temperature (±0.1°C) were measured in the sediment excavated by the crabs outside the burrow and in the upper 2 cm of the burrow water with a Sartorius ORP (redox) combination electrode and a WTW Sentix 41 pH-electrode connected to a WTW portable meter (Multi 340i), respectively. The above mentioned parameters were also measured in the sediment surrounding the burrow (from here on referred to as “burrow wall sediment” - BWS) at sediment depths of 1, 10, 30 and 50 cm. To be able to measure the reduction potential in deeper sediment depths, one half of the burrow was carefully opened. When the desired measuring depth inside the burrows was below the water table, the water was manually removed with a small vial before taking the measurement. Measurements beyond 50 cm sediment depth were technically too difficult due to the rapid refilling of the burrow with water from below. At each sediment depth, the electrodes were inserted perpendicularly to the burrow wall, thereby subsequent readings at horizontal distances of 2, 5, 8 and 15 cm from the BWS were obtained (Figure 3). As reduction potentials in the sediment around fiddler crab burrows have been observed as being influenced at a range of 1–4 cm [18], [19] and *U. cordatus* burrows are larger, we choose a larger maximal horizontal distance of 15 cm to insure that the change in reduction potential were within the measured radius.

In addition to the burrows, non-bioturbated sediment (referred to as the control) was also measured for comparison. Sediment cores were taken at least 15 cm away from burrows and stilt roots to sample areas where the influence of burrows and roots on the surrounding sediment was most likely low. A peat sampler (Eijkelkamp) of 50 cm length and 6 cm diameter was used to obtain the cores. Redox potential, pH and temperature of the sediment core were measured at depths of 1, 10, 30 and 50 cm.

Redox potential, temperature and pH values of each measurement were used to calculate the rH values [37]. These values are an indicator of the reduction force of a redox system and range between 0 (strongly reducing conditions) and 42 (strongly oxidizing conditions).

### Statistical analyses

The statistical analyses were carried out following protocols for data exploration and analysis of Zuur et al. [38], [39] using the statistical programming environment R [40] with the “nlme” [41] and the “ggplot2” [42] package. Data were checked for outliers (Cook distance) and these were, if necessary, removed. For information on data sets used for the analyses see Data S1.

Burrow entrance diameters, depth from the surface sediment to the water surface inside burrows, and rH values of burrow water and excavated sediment were each separately analyzed with a one way ANOVA for differences over time. When the trend over time was not linear, time was considered as a factor and a Tukey post-hoc test was applied to detect differences throughout the wet season.

Gaussian linear mixed-effects (LME) models were used for the following analyses, as they can account for inner variation between sediment cores or burrows [38], [43], [44]. The CO_2_ efflux rates of burrows (*F_b_*) and controls were analyzed with a LME model to test for differences over time. The random intercept of the model allowed for heterogeneity among burrows and between the sampling sites of plain sediment. To find the optimal set of fixed terms, a backwards model selection was used based on the maximum likelihood ratio test (ML) and/or on the Akaike Information Criterion (AIC). The validity of the model was checked by examination of diagnostic plots of model residuals. Final models were checked for homogeneity between residuals versus fitted values and covariates. Independence was examined by plotting residuals versus time. The final model was generated and evaluated with the restricted maximum likelihood estimation (REML) and these models are presented in Tables S1, S2, S3, S4, S5 and S6. All following LME models were analyzed as described above. Estimated values from statistical analyses are presented as mean ± standard error (se). Respiration rates of crabs were analyzed with a LME model to test for difference between treatments, number of measurements (from least acclimated to acclimated) and their respective interaction terms. The random intercept accounted for heterogeneity between individual crabs. No function for the temporal correlation structure was considered in the repeated measurements of the same crab, to be able to detect those differences between repeated measurements.

The rH data for the BWS were analyzed with a LME model to test for differences among sediment depths, horizontal distances from the burrow wall, time and their interaction terms. The random intercept accounted for heterogeneity among individual burrows. Control rH values were tested with a LME model for differences between sediment depths, time and their interaction term. The random term allowed for heterogeneity between sediment cores. Since general trends over time for the control and burrow rH data had already been analyzed in the two LME models and to keep the following models simple, control and burrow rH data were analyzed separately for each month. Monthly data sets of rH values from burrows and controls were analyzed in four LME models to test for differences among horizontal sampling levels, sediment depths and their interaction terms. BWS values of 2 (BWS2), 5 (BWS5), 8 (BWS8) and 15 cm (BWS15) and the control (>15 cm) represented the five horizontal sampling levels. BWS values and the control value were set as categorical covariates, because the exact horizontal distances of the control cores to the neighboring below ground burrow corridors was unknown. The random term allowed for heterogeneity among sampling locations (sediment core or burrow).

A Pearson correlation was applied to measure the relationship between surface rH values and CO_2_ efflux rates of the plain sediment. Because the CO_2_ efflux rate of a burrow could be associated to several rH values in the burrow wall sediment (from horizontal distances of 2 cm in several sediment depths) no correlation was performed.

## Results

### CO_2_ efflux rates

Burrow entrance diameters did not significantly differ over time (F = 3.3, df = 1, *p* = 0.07, Table 1) insuring that burrows of different samplings were comparable to each other. Depths from the sediment surface to the water surface inside burrows changed over time (F = 9.9, df = 1, *p*<0.001) and were largest in October (Tukey post-hoc: *p*≤ 0.002, Table 1). The mean burrow wall area exposed to air was 0.04±0.02 m^2^ (mean ± standard deviation, n = 78) and ranged from 0 (burrow completely filled with water) to 0.1 m^2^.

**Table 1:** Field data of measured and estimated parameters.

	2012							
	April		July		September		October	
	**Burrow entrance diameter (cm)**							
Mean ± se (n)	6.6±0.3	(24)	5.9±0.2	(24)	6.2±0.1	(19)	5.8±0.3	(11)
Minimum	4.1		3.4		5.3		4.6	
Maximum	9.6		7.4		7.4		7.4	
	**Burrow water level from the surface (cm)**							
Mean ± se (n)	16.2±0.9	(24)	21.7±2.9	(24)	18.3±1.3	(19)	33.8±2.2	(11)
Minimum	7		5		10		27	
Maximum	26		50		29		50	
	**CO_2_ efflux rate of plain sediment (µmol m^−2 ^s^−1^)**							
Mean ± se (n)	0.7±0.07	(88)	0.9±0.07	(96)	1.3±0.07	(93)	1.3±0.06	(96)
	**CO_2_ efflux rate of individual burrows (µmol burrow^−1 ^s^−1^)**							
BED: 5 cm, mean ± se (n)	0.1±0.01	(116)	0.2±0.02	(119)	0.1±0.01	(95)	0.1±0.01	(55)
BED: 6 cm, mean ± se (n)	0.2±0.01	(116)	0.3±0.03	(119)	0.1±0.01	(95)	0.2±0.02	(55)
BED: 7 cm, mean ± se (n)	0.3±0.02	(116)	0.4±0.04	(119)	0.2±0.01	(95)	0.2±0.03	(55)
	**Reduction potential of burrow water**							
Mean ± se (n)	16.2±0.9	(22)	16.8±0.5	(22)	18.0±0.9	(16)	16.2±0.7	(9)
	**Reduction potential of excavated sediment**							
Mean ± se (n)	15.5±0.8	(25)	16.1±0.7	(24)	16.0±0.7	(17)	17.5±0.9	(12)

Burrow entrance diameter, burrow water level from the surface, CO_2_ efflux rate of plain sediment and inhabited burrows with specific diameter, reduction potential of burrow water and excavated sediment.

Abbreviation: BED = burrow entrance diameter (in cm), n = sample size, se = standard error.

Examining the CO_2_ efflux rates of burrows (*F_b_*) for differences over time showed that the LME model which included the term time was not better than the model without this term (Likelihood Ratio/L. Ratio = 1.1, df = 1, *p* = 0.3, Table S1) indicating that measured values did not differ over time. By contrast, control sediment CO_2_ efflux rates increased over time (Table 1); removing the term time from the corresponding LME model led to a less suitable model, so the factor was retained (L. Ratio = 15.3, df = 1, *p*<0.001, Table S2). Calculated CO_2_ efflux rates of individual burrows ranged from 0.1 µmol burrows^−1 ^s^−1^ for burrows with a BED of 5 cm to 0.4 µmol burrows^−1 ^s^−1^ for burrows with a BED of 7 cm (Table 1).

### Respiration of *U. cordatus*

Mean carapace width and derived wet masses of crabs are given in Table 2. A LME model of the crab respiration data with the interaction term treatment×number of observations was significantly better than a model without this interaction (L. Ratio = 12.5, df = 1, *p*<0.001, Table S3). This indicates that crab respiration rates varied considerably between individuals of one treatment, but decreased during the five consecutive measurements. Crabs resting in seawater had the lowest respiration rates (Table 2). During the measurements, the least acclimated crabs produced foam in front of their mouthparts, whereas more acclimated crabs did not foam and stopped respiring for a few seconds up to several minutes. Respiration rates of individual crabs with a wet mass corresponding to burrow entrance diameters of 5, 6 and 7 cm, respectively, are presented for the “worst” (crab least acclimated, resting in air) and “best” case scenarios (crab with the longest acclimatization time and resting in water) in Table 2. The crabs’ contribution to the CO_2_ efflux rates of individual burrows was 55–100% in the “worst” case and 20–60% in the “best” case scenario (Table 1, Table 2).

**Table 2:** Data of *U. cordatus* respiration measurements.

	Crab in air		Crab in seawater	
	**Carapace width (cm)**			
Mean ± se (n)	7.3±0.1	(14)	7.0±0.1	(14)
Median	7.4		7.0	
Minimum	6.5		6.2	
Maximum	7.8		7.5	
	**Wet mass (g)**			
Mean ± se (n)	161.6±6.3	(14)	140.4±5.9	(14)
Median	162.4		137.7	
Minimum	113		98.2	
Maximum	193.5		172.4	
	**Crab respiration (µmol CO_2 _kg^−1 ^s^−1^)**			
Mean ± se (n) of measurement 1	2.3±0.4	(13)	1.4±0.2	(13)
Mean ± se (n) of measurement 2	1.4±0.2	(13)	1.0±0.2	(12)
Mean ± se (n) of measurement 3	1.1±0.1	(13)	0.9±0.2	(12)
Mean ± se (n) of measurement 4	0.9±0.1	(12)	0.7±0.1	(12)
Mean ± se (n) of measurement 5	0.9±0.1	(12)	0.8±0.2	(12)
Minimum of all measurement (n)	0.4	(63)	0.04	(61)
Maximum of all measurement (n)	4.1	(63)	2.4	(61)
	**Crab respiration - worst case (µmol CO_2_ crab^−1 ^s^−1^)**			
BED: 5 cm, WM: 28 g, mean ± se (n)	0.11±0.02	(13)	0.07±0.01	(13)
BED: 6 cm, WM 55 g, mean ± se (n)	0.19±0.03	(13)	0.12±0.02	(13)
BED: 7 cm, WM 93 g, mean ± se (n)	0.30±0.05	(13)	0.19±0.02	(13)
	**Crab respiration - best case (µmol CO_2_ crab^−1 ^s^−1^)**			
BED: 5 cm, WM: 28 g, mean ± se (n)	0.04±0.004	(12)	0.04±0.01	(12)
BED: 6 cm, WM 55 g, mean ± se (n)	0.07±0.01	(12)	0.06±0.01	(12)
BED: 7 cm, WM 93 g, mean ± se (n)	0.12±0.01	(12)	0.10±0.02	(12)

Carapace width, estimated wet mass after Diele [36], average respiration rates during the five measurements and respiration rate of individual crabs of a specific wet mass inhabiting burrows with different burrow entrance diameters for the “worst” case scenario (crab not acclimated, values calculated from measurement 1) and for the “best” case scenario (crab with longest acclimatization time, values calculated from measurement 5).

Abbreviation: BED = burrow entrance diameter (in cm), n = sample size, se = standard error, WM = wet mass.

### Estimates for CO_2_ release of mangrove sediment

**Figure 4:**
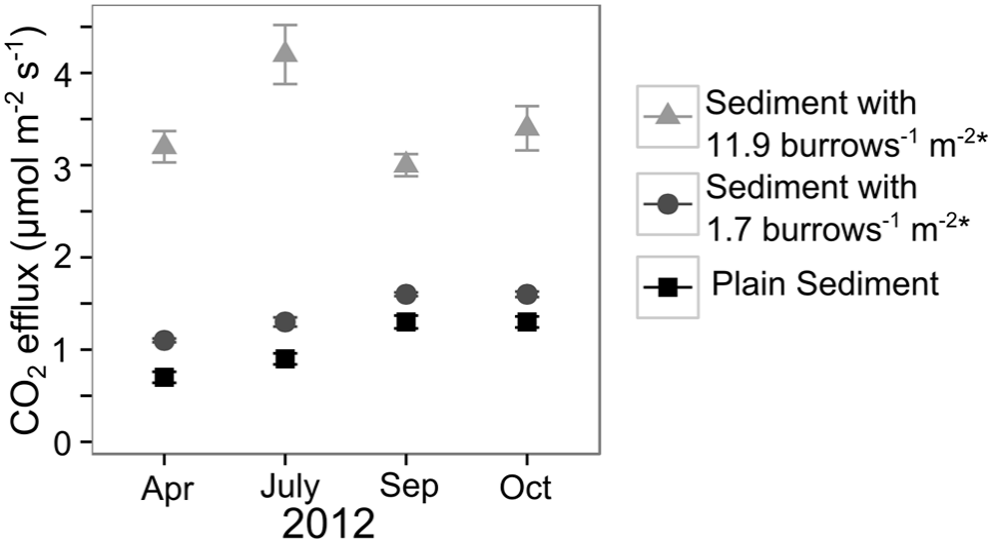
CO_2_ efflux rates (µmol m^−2 ^s^−1^) of plain sediment and sediment with a burrow density of 1.7 and 11.9 burrows^−1 ^m^−2^. *For these estimates all burrow entrance diameters were set at 6 cm.

Calculated CO_2_ efflux rate estimates of mangrove sediment with 1.7 and 11.9 burrows^−1 ^m^−2^ increased over time. The reason for this was an increase in CO_2_ efflux rates of plain sediment over time due to seasonality, while individual burrow CO_2_ efflux rates remained constant over time (Figure 4). Estimates of CO_2_ efflux rate of sediment with *U. cordatus* burrows (Table 3, Figure 4) are 15 to 500% (considering 1.7 and 11.9 burrows^−1 ^m^−2^, respectively) higher than for plain sediment (Table 3). Despite the small contribution of burrow openings to total sediment surface area (<5%), *U. cordatus* burrows, especially in densely rooted sediment, can increase the CO_2_ efflux rate of plain sediment by 92 to 500% (Table 3, Figure 4).

**Table 3:** CO_2_ efflux rates of mangrove sediment.

	2012							
	April		July		September		October	
Density: 1.7 burrows^−1 ^m^−2^	**CO_2_ efflux rate of mangrove sediment (µmol m^−2 ^s^−1^)**							
BED: 5 cm, mean ± se (n)	1.0±0.02	(116)	1.2±0.03	(119)	1.5±0.01	(95)	1.5±0.02	(55)
BED: 6 cm, mean ± se (n)	1.1±0.02	(116)	1.3±0.05	(119)	1.6±0.02	(95)	1.6±0.03	(55)
BED: 7 cm, mean ± se (n)	1.2±0.03	(116)	1.5±0.06	(119)	1.7±0.02	(95)	1.7±0.05	(55)
Density: 11.9 burrows^−1 ^m^−2^	**CO_2_ efflux rate of mangrove sediment (µmol m^−2 ^s^−1^)**							
BED: 5 cm, mean ± se (n)	2.4±0.12	(116)	3.2±0.22	(119)	2.5±0.08	(95)	2.7±0.17	(55)
BED: 6 cm, mean ± se (n)	3.2±0.17	(116)	4.2±0.32	(119)	3.0±0.12	(95)	3.4±0.24	(55)
BED: 7 cm, mean ± se (n)	4.1±0.23	(116)	5.4±0.44	(119)	3.6±0.16	(95)	4.1±0.33	(55)
Density: 1.7 burrows^−1 ^m^−2^	**Increase of CO_2_ efflux rate to plain sediment (%)**							
BED: 5 cm (AP: 0.3%)	43		33		15		15	
BED: 6 cm (AP: 0.5%)	57		44		23		23	
BED: 7 cm (AP: 0.7%)	71		67		31		31	
Density: 11.9 burrows^−1 ^m^−2^	**Increase of CO_2_ efflux rate to plain sediment (%)**							
BED: 5 cm (AP: 2.3%)	243		256		92		108	
BED: 6 cm (AP: 3.4%)	357		367		131		162	
BED: 7 cm (AP: 4.6%)	486		500		177		215	

Rates and the increase in % compared to plain sediment for average burrow densities of 1.7 burrows^−1 ^m^−2^ for *Rhizophora mangle* dominated areas at the Furo Grande [30] and 11.9 burrows^−1 ^m^−2^ for densely rooted areas around *R. mangle* trees.

Abbreviation: AP = areal proportion of the burrow opening per m^2^, BED = burrow entrance diameter (in cm), n = sample size, se = standard error, WM = wet mass.

### Reduction potential

**Figure 5:**
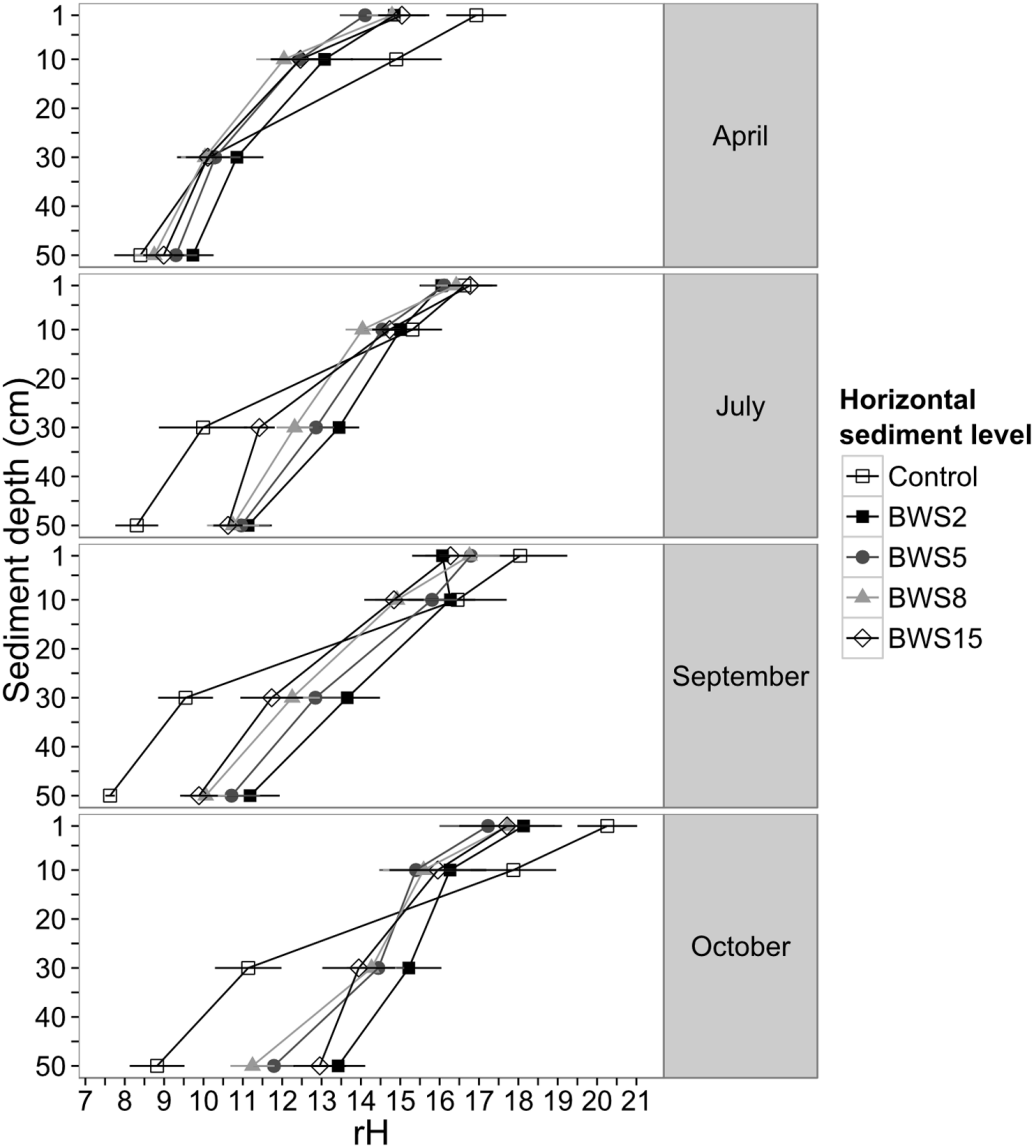
Burrow and plain sediment rH values for each sampling campaign in the year 2012. Mean sediment rH values ± standard error (se) plotted against sediment depth (cm) (sample size (n) for each month: April: n = 308, July: n = 363, September: n = 282 and October: n = 240). Different grey shades and symbols represent the different horizontal sampling distances from the burrow wall sediment (BWS, BWS2 = 2 cm, BWS5 = 5 cm, BWS8 = 8 cm, BWS15 = 15 cm and control >15 cm).

The rH values of both burrow water (F = 0.6, df = 1, *p* = 0.4, Table 1) and excavated sediment (F = 2.3, df = 1, *p* = 0.1, Table 1) did not differ over time. The final model of the BWS rH data indicates that rH values decreased with sediment depth and horizontal distance as well as over time (Figure 5); a LME model of the BWS rH data with a three way interaction term was not significantly better than a model without this interaction (L. Ratio = 4.4, df = 9, *p* = 0.9, Table S4). Furthermore, the interaction terms horizontal depth×time (L. Ratio = 1.0, df = 3, *p* = 0.8, Table S4) and sediment depth×time (L. Ratio = 3.2, df = 3, *p* = 0.4, Table S4) were eliminated. The model with the interaction term horizontal distance×sediment depth was better than without interaction (L. Ratio = 17.2, df = 9, *p* = 0.045, Table S4). However, we continued with the simpler (using fewer degrees of freedom) and slightly better model (AIC of model with the interaction term: 4389.1 and without interaction term: 4388.4) by excluding the interaction term. Finally, comparing models where each main term was excluded once with the original model containing all three main terms showed that the latter was best (sediment depth: L. Ratio = 555.0, df = 3, *p*<0.001; horizontal distance: L. Ratio = 35.6, df = 3, *p*<0.001; time: L. Ratio = 20.7, df = 1, *p*<0.001, Table S4).

The model of the control rH data with the interaction term sediment depth×time was significantly better (L. Ratio = 10.1, df = 1, *p* = 0.0015, Table S5) than the model without this term indicating that control rH values decreased with sediment depth, but had different depth-rH curves over time (Figure 5). Further, the surface rH values positively correlated with the CO_2_ efflux rates of plain sediment (correlation coefficient = 0.18, *p*<0.001).

In the next step, BWS and control rH data were compared separately for each sampling campaign. The comparison of the four models including and excluding the interaction term sediment depth×time showed that the interaction term significantly improved the four models (April: L. Ratio = 18.0, df = 4, *p* = 0.001, July: L. Ratio = 33.2, df = 4, *p*<0.001, September: L. Ratio = 38.3, df = 4, *p*<0.001, October: L. Ratio = 44.0, df = 4, *p*<0.001, Table S6). Hence, at any point of time rH values decreased with increasing sediment depth, and depth-rH curves differed between horizontal sampling levels, in the way that rH values decreased mostly with increasing horizontal distances (Figure 5). Measured pH values are not shown in detail; they ranged from 3.5 to 7.1 for plain sediment and from 4.3 to 7.8 for burrow wall sediment.

## Discussion

Measured CO_2_ efflux rates of individual burrows are composed of three elements: The respiration of the inhabiting crab itself, the CO_2_ released by the burrow wall sediment and the CO_2_ released by the water inside the burrow.

### The crabs’ contribution to the CO_2_ efflux rate of burrows

In our study measurements of CO_2_ exhaled by isolated crabs showed that high CO_2_ production by respiration was related to stress. Crabs in our study, particularly those kept without seawater in the PVC basin, were probably not fully acclimated during the 50 min respiration measurements, because CO_2_ respiration of crabs resting in air were always higher in the five measurements compared to crabs resting in water (Table 2). Some crabs exhaled four or fewer times in 10 min (see minimum values in Table 2) suggesting reasonable acclimatization.

During field measurements, crabs were sometimes seen inside their burrows when the respiration chamber was opened between measurements. The crabs did not appear to be stressed by the chamber, since they moved slowly and did not produce foam, as they do when they are handled or otherwise stressed [45]. Therefore, respiration rates of *U. cordatus* measured in the laboratory for the “worst” case scenario (crabs with no acclimatization time and resting in air) are probably overestimating the crabs’ respiration under field conditions. Respiration rates for the “best” case scenario (crabs with longest acclimatization time resting in water) on the other hand, probably resemble field conditions more closely. Our data thus suggest that crabs contributed about 20–60% to the CO_2_ efflux rates of individual burrows, which leaves the burrow CO_2_ efflux rates without crabs to range between 0.04–0.32 µmol burrows^−1 ^s^−1^ for burrows of 5 to 7 cm entrance diameter (Table 1). Respiration rates of an acclimated *U. cordatus* specimen (250 g wet mass, in seawater) are 0.19±0.04 µmol CO_2_ crab^−1 ^s^−1^ and are similar to rates recorded for a large sesarmid crab (*Neoepisesarma versicolor*, 250 g wet mass, in seawater) of 0.24 µmol CO_2_ crab^−1 ^s^−1^ [46].

### CO_2_ efflux rates of burrows and sediment

CO_2_ efflux rates of inhabited *U. cordatus* burrows were at least 20 times higher (Table 1) than estimated rates from inhabited fiddler crab burrows (0.005 µmol burrows^−1 ^s^−1^) measured in a Tanzanian mangrove forest [24]. Higher CO_2_ efflux rates of *U. cordatus* burrows are probably caused by the larger body size and higher respiration of the crab and the corresponding larger burrow size (and depth) compared with *Uca* spp. and their burrows (average wet mass of *Uca* spp.: 3.2 g, 0.002 µmol CO_2_ crab^−1 ^s^−1^, average burrow entrance diameter not reported, [24]). Mean CO_2_ efflux rates of plain sediment in our study ranged from 0.7 to 1.3 µmol m^−2 ^s^−1^ and were similar to other mangrove sediments [20], [22], e.g. to values measured by Lovelock [47] for sites in the Caribbean, Australia and New Zealand, which ranged from −0.25 to 2.97 µmol m^−2 ^s^−1^. In line with our first hypothesis, CO_2_ efflux rates of mangrove sediment containing burrows inhabited by *U. cordatus* were 15 to 500% higher than for plain sediment depending on burrow density and month (Table 3). In a Tanzanian mangrove forest, Kristensen et al. [24] recorded CO_2_ efflux rates of sediment with inhabited *Uca* spp. burrows in areas without obvious above ground roots. Measured rates ranged between 0.68 µmol m^−2 ^s^−1^ (68±17 burrows^−1 ^m^−2^) and 1.38 µmol m^−2 ^s^−1^ (237±53 burrows^−1 ^m^−2^) and were similar to ours with 1.0±0.02 until 1.7±0.05 µmol m^−2 ^s^−1^ based on an average *U. cordatus* burrow density of 1.7 burrows^−1 ^m^−2^ (Table 3). Although CO_2_ release of individual fiddler crab burrows is much lower than that of *U. cordatus*, the burrow density of fiddler crabs at the Tanzanian study site was much higher (68–626 burrows^−1 ^m^−2^, [24]) than burrow densities of *U. cordatus* recorded near our study site (1.7 burrows^−1 ^m^−2^ at an average, Diele et al. [30]). Thus, a high density of burrows may compensate for a lower per-burrow CO_2_ efflux rate.

In addition to *U. cordatus* burrows, *Uca* spp. burrows were also present at our study site (28 to 105 burrows^−1 ^m^−2^ with an average of 57±18 burrows^−1 ^m^−2^, mean±standard deviation, n = 42, with each sample area 1 m^2^, unpublished data). Future investigations should focus on the entire crab community of a mangrove site, involving all burrowing crabs, to obtain more precise estimates of overall CO_2_ efflux rates from the mangrove sediment.

The amount of CO_2_ released by sediment or burrows depends on the prevalence of organic carbon in the sediment and its oxidation pathways by microorganisms [48]–[50]. In a study on the Ajuruteua peninsula the organic matter content and mean cell numbers of bacteria were measured in the BWS of *U. cordatus* burrows (70 cm depth) and were similar to corresponding values of the surface sediment [51]. Therefore, microbial activity in BWS may be more similar to surface sediment than to non-bioturbated sediment at 70 cm sediment depth. However, CO_2_ released from burrows may not exclusively originate from carbon oxidation in the BWS, but also from the carbon stock in deeper sediment layers which may be released via the burrows [20], [24]. Furthermore, the amount of CO_2_ released from the water accumulated inside the burrow is unknown. The water may contain CO_2_ originating from bacterial, meio- and macrofaunal respiration and from tidal input. How the bicarbonate system of the burrow water is constituted is also an unknown, e.g. how such systems react to increasing/decreasing CO_2_ concentrations and whether changing water levels inside burrows influence the burrow water CO_2_ gas exchange. CO_2_ dissolved in water may also be partly exported by the groundwater during the ebb tide [52].

Apart from microbial decomposition in the sediment, CO_2_ is produced by root respiration. Information about root respiration of mangrove tree species is scarce; root respiration rates are reported to be low for *R. mangle* trees. Kristensen et al. [24] estimated the amount of CO_2_ released by pneumatophores of *Sonneratia alba* and *Avicennia marina* in a Tanzanian mangrove forest, yielding an equivalent or higher efflux rate than sediment with burrows. Due to technical reasons we had to avoid measuring near aboveground aerial roots. However, underground root respiration may have contributed to the CO_2_ released by burrows and, equally, by surface sediment without burrows. As *U. cordatus* prefers areas with *R. mangle* roots when constructing its burrows [31], their burrows possibly function as an exhalant channel for CO_2_ produced by root respiration. To test this hypothesis, further work on CO_2_ release must overcome technical restrictions and focus on areas where crabs and mangrove tree roots occur closely together.

### Temporal and spatial patterns of reduction potentials

Reduction potentials of the burrow water and the excavated sediment were similar during the wet season and relatively oxidized compared to the BWS and control rH values in deeper sediment depths. This indicates that burrow water is regularly mixed with more oxidized water or oxygen by, for example, tidal flushing or groundwater flow [53]–[55]. Further, excavated sediment oxidizes at the surface when in contact with atmospheric oxygen. Thus, bioturbation by crabs significantly oxidizes previously reduced sediment.

In contrast to the stable values in the burrow water and the excavated sediment, rH values in the BWS changed over time. In April, BWS had the lowest rH values. At 1 and 10 cm sediment depth rH values were even lower compared to plain sediment. The rising water column inside the burrows (Table 1) and waterlogging of the sediment caused by the high precipitation rates in this month may have led to anoxic conditions along the burrow wall. In contrast, during the last sampling at the end of the wet season rain showers, temporally filling up burrows became less frequent and water levels sank to 50 cm below the surface (Table 1). The longer exposure of BWS to air at the end of the wet season led to more oxidized conditions at 30–50 cm sediment depths compared with plain sediment. Additionally, from July onwards BWS rH and control rH values increased with decreasing precipitation rates (Figure 2, Figure 5).

The spatial pattern of the *U. cordatus* BWS rH values indicated a wider oxidized zone, reaching up to 15 cm away from the burrow, compared to the *Uca sp.* burrows, where the oxidized zone reached only 1.5–4 cm into the surrounding sediment [18], [19]. This difference is most likely due to the much larger size and depth of *U. cordatus* burrows, leading to improved solute exchange conditions between the BWS and tidal water, groundwater and atmospheric air compared to the smaller fiddler crab burrows. Why rH values for the up to 15 cm horizontal distance from the burrow wall did not approximate control values remains unclear. However, an approximation may have been masked by the high variance of the rH values due to nearby below ground burrow galleries or roots [56], which could not be seen during the measurements. Since rH values in the BWS were not always more oxidized compared to plain sediment we reject our second hypothesis. The reduction potential of the sediment is further affected by different environmental and biotic factors such as water logging, tree roots and age of the forest stand [1], [18], [19], [48], [49], [57].

### Temporal pattern in CO_2_ release

CO_2_ efflux rates of plain surface sediment increased over time. Possibly surface sediment became more oxidized towards the end of the wet season and a change in the microbial decomposition process may thus have resulted in the different CO_2_ efflux rates [22]. Another possible reason could be that CO_2_, which is normally released over the sediment surface, may have been dissolved after rainfalls and stored in rain water filling sediment pore space. This would have increased the sediment CO_2_ concentrations, while less CO_2_ would have been released from the sediment, as observed in our study at the beginning of the wet season. Corresponding results have been obtained by studies performed in a temperate pinewood forest [58], [59]. In contrast to the CO_2_ efflux rates of plain surface sediment, the CO_2_ efflux rates of individual burrows did not change over time, despite simultaneously varying rH values. The reasons for this are not clear.

## Conclusions

Until now, the ecological role of *Ucides cordatus* has been mostly discussed in terms of carbon retention through litter feeding. The results of the present study emphasize the crabs’ importance for carbon export via CO_2_ release. Respiration by *U. cordatus* and CO_2_ release by its burrows increase the CO_2_ efflux rate compared to plain sediment by 15–71% assuming a conservative average density of 1.7 burrows^−1 ^m^−2^ for *Rhizophora mangle* dominated forests at the Caeté estuary in North Brazil. This increase in CO_2_ efflux rate is particularly high considering the relatively low proportion (0.3–0.7%) for which crab burrow entrances of *U. cordatus* account for per sediment area. Furthermore, burrows lead to changes in the reduction potential in the sediment and may thereby indirectly influence microbial decomposition pathways and CO_2_ release. This study further demonstrates that the reduction potential around the burrow walls underlies spatio-temporal variations which need to be considered to improve estimates of CO_2_ release from mangrove sediments. Future studies should disentangle carbon release and carbon storage by crabs and determine whether these two processes are in balance. Moreover, we underline the importance of including CO_2_ efflux rates of burrows and respiration rates of crabs into carbon budget estimates of mangrove ecosystems.

## Supporting Information

Table S1 **Final linear mixed-effects model of burrow CO_2_ efflux rate data.**
(PDF)

Table S2 **Final linear mixed-effects model of control CO_2_ efflux rate data.**
(PDF)

Table S3 **Final linear mixed-effects model of crab respiration data.**
(PDF)

Table S4 **Final linear mixed-effects model of burrow rH data.**
(PDF)

Table S5 **Final linear mixed-effects model of control rH data.**
(PDF)

Table S6 **Final linear mixed-effects model of control-burrow rH data.**
(PDF)

Data S1 **Complete data set used for statistical analyses.**
(PDF)
