# Supplementary material for: Burrows of the Semi-Terrestrial Crab Ucides cordatus Enhance CO2 Release in a North Brazilian Mangrove Forest
Source: PLoS One. 2014 Oct 14;9(10):e109532. doi: 10.1371/journal.pone.0109532 (PMC4196909; doi:10.1371/journal.pone.0109532)
Supplement: Table S1 — Final linear mixed-effects model of burrow CO2 efflux rate data. (PDF) [file pone.0109532.s001.pdf]

Table S1: Final linear mixed-effects model of burrow CO_2 efflux rate data

The final optimal model was selected after a stepwise backwards model selection using the likelihood ratio test:

$$\text{Burrow}\,\text{CO}_2\ \text{efflux rate}_{ib} \sim \alpha + 1 + \alpha_b + \varepsilon_{ib}, \varepsilon_{ib} \sim N(0, \sigma^2)$$

Burrow CO_2 efflux $rate_{ib}$ is the rate of observation *i* for burrow *b*, where *b* runs from 1 to 86, and *i* is the observation for each burrow that ranges from 1 to 4 (number of samplings over time). The final model above means that CO_2 efflux rate is modelled with an intercept represented by α and a slope of 1. The term a_b is the random effect representing the between-burrow variation and is significant (L. Ratio = 606.0, df = 1, $p < 0.001$). The unexplained variance ε is assumed to be normally distributed with mean 0 and variance σ^2.
