# Supplementary material for: Burrows of the Semi-Terrestrial Crab Ucides cordatus Enhance CO2 Release in a North Brazilian Mangrove Forest
Source: PLoS One. 2014 Oct 14;9(10):e109532. doi: 10.1371/journal.pone.0109532 (PMC4196909; doi:10.1371/journal.pone.0109532)
Supplement: Table S2 — Final linear mixed-effects model of control CO2 efflux rate data. (PDF) [file pone.0109532.s002.pdf]

Table S2: Final linear mixed-effects model of control CO_2 efflux rate data

The final optimal model was selected after a stepwise backwards model selection using the likelihood ratio test:

$$\text{Control}\,\text{CO}_2 \text{ efflux rate}_{is} \sim \alpha + \text{Time}_{is} + \alpha_b + \varepsilon_{is}, \varepsilon_{is} \sim N(0, \sigma^2)$$

Control CO_2 efflux rate_{is} is the rate of observation *i* for sampling point *s*, where *c* runs from 1 to 96, and *i* is the observation for each sampling point that ranges from 1 to 4 (number of samplings over time). The final model above means that the control CO_2 efflux rate is modelled as a function of time. Time is a categorical covariate. The term a_b is the random effect representing the between-sampling point variation and is significant (L. Ratio = 208.0, df = 1, $p < 0.001$). The unexplained variance ε is assumed to be normally distributed with mean 0 and variance σ^2. The intercept of the model is represented with α.
