# Supplementary material for: Burrows of the Semi-Terrestrial Crab Ucides cordatus Enhance CO2 Release in a North Brazilian Mangrove Forest
Source: PLoS One. 2014 Oct 14;9(10):e109532. doi: 10.1371/journal.pone.0109532 (PMC4196909; doi:10.1371/journal.pone.0109532)
Supplement: Table S3 — Final linear mixed-effects model of crab respiration data. (PDF) [file pone.0109532.s003.pdf]

Table S3: Final linear mixed-effects model of crab respiration data

The final optimal model was selected after a stepwise backwards model selection using the likelihood ratio test:

$$\text{Crab respiration}_{ij} \sim \alpha + \text{Treatment}_{ij} + \text{Measurement}_{ij} + \text{Treatment}_{ij} \times \text{Measurement}_{ij} + \alpha_j + \varepsilon_{ij},$$
$$\varepsilon_{ij} \sim N(0, \sigma^2)$$

Crab respiration$_{ij}$ is the rate of observation *i* for an individual crab *j*, where *j* runs from 1 to 28, and *i* is the observation for each individual crab that ranges from 1 to 5. The final model above means that crab respiration is modelled as a function of treatment, (number of) measurement and their interaction term. Treatment is a categorical covariate and observation a continuous. The term a_b is the random effect representing the between-crabs variation and is significant (L. Ratio = 65.0, df = 1, p < 0.001). The unexplained variance ε is assumed to be normally distributed with mean 0 and variance σ^2. The intercept of the model is represented with α.
