# Supplementary material for: Burrows of the Semi-Terrestrial Crab Ucides cordatus Enhance CO2 Release in a North Brazilian Mangrove Forest
Source: PLoS One. 2014 Oct 14;9(10):e109532. doi: 10.1371/journal.pone.0109532 (PMC4196909; doi:10.1371/journal.pone.0109532)
Supplement: Table S4 — Final linear mixed-effects model of burrow rH data. (PDF) [file pone.0109532.s004.pdf]

Table S4: Final linear mixed-effects model of burrow rH data

The final optimal model was selected after a stepwise backwards model selection using the likelihood ratio test:

$$\text{Burrow rH}_{ib} \sim \alpha + \text{Horizontal distance}_{ib} + \text{Sediment depth}_{ib} + \text{Time}_{ib} + \alpha_b + \varepsilon_{ib}, \varepsilon_{ib} \sim N(0, \sigma_d^2)$$

Burrow rH_{ib} is the observation *i* for each burrow *b*, where *b* runs from 1 to 86, and *i* is the observation for each burrow that ranges from 1 to 4 (number of samplings over time). The final model above means that burrow rH data is modelled as a function of horizontal distance, sediment depth and time. Horizontal depth, sediment depth are categorical covariates and time is a continuous covariate. The term a_b is the random effect representing the between-burrow variation and is significant (L. Ratio = 375.8, df = 1, $p < 0.001$). The unexplained variance ε_{ib} is assumed to be normally distributed with mean 0 and variance σ_d^2 where d takes 4 different values according to each sediment depth level. The intercept of the model is represented with α.
