# Supplementary material for: Burrows of the Semi-Terrestrial Crab Ucides cordatus Enhance CO2 Release in a North Brazilian Mangrove Forest
Source: PLoS One. 2014 Oct 14;9(10):e109532. doi: 10.1371/journal.pone.0109532 (PMC4196909; doi:10.1371/journal.pone.0109532)
Supplement: Table S5 — Final linear mixed-effects model of control rH data. (PDF) [file pone.0109532.s005.pdf]

Table S5: Final linear mixed-effects model of control rH data

The final optimal model was selected after a stepwise backwards model selection using the likelihood ratio test:

$$\text{Control rH}_{ic} \sim \alpha + \text{Sediment depth}_{ic} + \text{Time}_{ic} + \text{Sediment depth}_{ic} \times \text{Time}_{ic} + \alpha_c + \varepsilon_{ic}, \varepsilon_{ic} \sim N(0, \sigma_d^2)$$

Control rH_{ic} is the observation *i* for each sampling core *c*, where *c* goes from 1 to 48 and *i* is the observation for each core that ranges from 1 to 4 (number of samplings over time). The final model above means that the control rH is modelled as a function of sediment depth, time and their interaction. Time and sediment depth are continuous covariates. The term a_c is a random effect representing the between-core variation and was significant (L. Ratio = 30.4, df = 1, $p < 0.001$). The unexplained variance ε_{ic} is assumed to be normally distributed with mean 0 and variance σ_d^2 where d takes 4 different values according to each sediment depth level. The intercept of the model is represented with α.
