# Supplementary material for: Burrows of the Semi-Terrestrial Crab Ucides cordatus Enhance CO2 Release in a North Brazilian Mangrove Forest
Source: PLoS One. 2014 Oct 14;9(10):e109532. doi: 10.1371/journal.pone.0109532 (PMC4196909; doi:10.1371/journal.pone.0109532)
Supplement: Table S6 — Final linear mixed-effects model of control-burrow rH data. (PDF) [file pone.0109532.s006.pdf]

Table S6: Final linear mixed-effects model of control-burrow rH data

Four LME models for the control-burrow rH data of each month were evaluated. Since the optimal fixed terms were similar for all four models, exemplary one model is presented below. The final optimal model was selected after a stepwise backwards model selection using the likelihood ratio test:

$$\text{Control-burrow rH}_{ip} \sim \alpha + \text{Treatment}_{ip} + \text{Sediment depth}_{ip} + \text{Treatment}_{ip} \times \text{Sediment depth}_{ip} + \alpha_p + \varepsilon_{ip},$$
$$\varepsilon_{ip} \sim N(0, \sigma_d^2)$$

Control and burrow rH_{ip} is the observation *i* for each sampling point, where *p* ranges from 1 to 86 for the burrow rH values and from 1 to 48 for the control values and *i* is the observation at each sampling point that is 1 (number of samplings over time). The final model above means that the control and burrow rH data is modelled as a function of treatment, sediment depth, and their interaction term. Treatment is a categorical covariate and sediment depth a continuous one. The term a_p is a random effect representing the between-sampling point variation and was significant (July: L. Ratio = 132.2, df = 1, $p < 0.001$, August: L. Ratio = 94.2, df = 1, $p < 0.001$, September: L. Ratio = 92.3, df = 1, $p < 0.001$, October: L. Ratio = 67.7, df = 1, $p < 0.001$). The unexplained variance ε_{ip} is assumed to be normally distributed with mean 0 and variance σ_d^2 where d takes 4 different values according to each sediment depth level (only the LME model for the month October had a variance of σ^2). The intercept of the model is represented with α.
