# Supplementary material for: Burrows of the Semi-Terrestrial Crab Ucides cordatus Enhance CO2 Release in a North Brazilian Mangrove Forest
Source: PLoS One. 2014 Oct 14;9(10):e109532. doi: 10.1371/journal.pone.0109532 (PMC4196909; doi:10.1371/journal.pone.0109532)
Supplement: Data S1 — Complete data set used for statistical analyses. (PDF) [file pone.0109532.s007.pdf]

Data S1: Complete data set used for statistical analyses

- Data set of distance of water inside burrow to surface and the burrow diameter (p.2)
- Data set of rH values of burrow water (p. 5)
- Data set of rH values of excavated sediment (p. 7)
- Data set of CO_2 efflux rates of burrows (F_b) (p. 9)
- Data set of control CO_2 efflux rates (plain sediment) (p. 17)
- Data set of carapace widths of *Ucides cordatus* (p. 29)
- Data set of crab respiration (p. 30)
- Data set of rH values in the burrow wall sediment (BWS) (p. 33)
- Data set of rH values in plain sediment (control) (p. 64)

Data set of distance of water inside burrow to surface and the burrow diameter

Crab burrow	Time	Distance of water inside burrow to surface (cm)	Burrow diameter (cm)
1	April	17	9.55
2	April	14	7.5
3	April	14	4.95
4	April	14	6.75
5	April	7.5	5
6	April	7	9.05
7	April	14	4.05
8	April	14	6.1
9	April	19	7.45
10	April	12	5.9
11	April	13	6.55
12	April	14	6.95
13	April	26	6.05
14	April	17	5.4
15	April	17	5.45
16	April	20	4.9
17	April	23	5
18	April	20	6.85
19	April	21	6.8
20	April	20	8.65
21	April	17	7.1
22	April	10	9.4
23	April	20	6.05
24	April	18	7
25	April	NA/0 floor completely covered by rain	NA
26	April	NA/0 floor completely covered by rain	NA
27	April	NA/0 floor completely covered by rain	NA
28	April	NA/0 floor completely covered by rain	NA
29	April	NA/0 floor completely covered by rain	NA
30	April	NA/0 floor completely covered by rain	NA

31	July	11	5.4
32	July	5	5
33	July	7	5.15
34	July	10	5.65
35	July	10	5.6
36	July	13	4.85
37	July	7	7
38	July	8	6
39	July	13	3.35
40	July	18	5.5
41	July	17	6.5
42	July	21	6.1
43	July	21	5.45
44	July	21	6.15
45	July	19	6.25
46	July	21	6.3
47	July	17	6.05
48	July	17	6.9
49	July	45	7.35
50	July	50	5.35
51	July	37	6.95
52	July	47	5.95
53	July	45	7.35
54	July	40	5.55
55	September	13	6.5
56	September	10	6.4
57	September	11	6.25
58	September	13.5	6.6
59	September	11	5.8
60	September	18	5.8
61	September	11	7.35
62	September	12	5.5
63	September	23	6.35

64	September	22	6.65
65	September	22	5.25
66	September	18	5.9
67	September	24	6.55
68	September	NA	NA
69	September	29	6.6
70	September	24	6.1
71	September	23	6.45
72	September	20	6.3
73	September	24	5.5
74	September	20	5.65
75	October	30	7.35
76	October	27	6.9
77	October	29	6.75
78	October	30	7.25
79	October	32	5.8
80	October	32	5.55
81	October	31	5.5
82	October	35	4.55
83	October	46	4.9
84	October	50	4.8
86	October	30	4.65

Data set of rH values of burrow water

Crab burrow	Time	rH
1	April	16.65
2	April	11.46
3	April	8.67
4	April	9.53
5	April	14.14
6	April	14.7
7	April	16.66
9	April	14.72
13	April	23.45
15	April	14.32
16	April	17.56
17	April	24.29
18	April	15.74
19	April	9.36
21	April	17.61
22	April	18.1
25	April	18.77
26	April	15.69
27	April	18.6
28	April	17.4
29	April	20.39
30	April	18.81
31	July	17.87
32	July	17.46
33	July	17.12
34	July	17.76
35	July	17.2
36	July	17.01
37	July	18.76
38	July	18.24
39	July	16.92
40	July	23.32
41	July	16.19
42	July	16.61
44	July	17.61
45	July	15.84
46	July	16.13
47	July	18.95
48	July	15.71
49	July	18.61
50	July	13.89
51	July	10.96
52	July	12.66
54	July	15.61
55	September	20.45
56	September	17.37
57	September	16.55
58	September	14.99
59	September	20.09

60	September	11.35
61	September	18.79
62	September	17.93
63	September	17.04
64	September	18.8
65	September	17.33
66	September	18.32
67	September	28.3
69	September	15.45
70	September	18.22
71	September	17.55
76	October	17.25
77	October	18.46
78	October	15.48
79	October	13.35
80	October	15.12
81	October	13.77
82	October	14.53
83	October	19.39
86	October	18.24

Data set of rH values of excavated sediment

Crab burrow	Time	rH
1	April	16.89
2	April	9.57
3	April	8.68
4	April	10.38
5	April	20.61
6	April	14.9
7	April	16.97
8	April	25.8
9	April	15.77
13	April	13.11
14	April	14.58
15	April	14.65
16	April	17.74
17	April	19.22
18	April	14.53
19	April	11.97
20	April	15.85
21	April	15.52
22	April	17.64
25	April	18.94
26	April	13.85
27	April	10.6
28	April	12.82
29	April	15.26
30	April	21.19
31	July	16.28
32	July	15.48
33	July	10.36
34	July	15.27
35	July	11.13
36	July	15.92
37	July	24.64
38	July	14.03
39	July	17.48
40	July	17
41	July	14.55
42	July	12.63
43	July	15.2
44	July	20.26
45	July	15.78
46	July	17.35
47	July	13.68
48	July	15.31
49	July	15.19
50	July	20.65
51	July	14.46
52	July	24.34
53	July	13.16
54	July	17.08

55	September	18.91
56	September	18.35
57	September	16.29
58	September	16.38
59	September	20.61
60	September	13.01
61	September	16.96
62	September	13.91
63	September	15.74
64	September	15.47
65	September	20.45
66	September	10.03
67	September	15.1
68	September	12.5
69	September	16.96
70	September	16.65
71	September	14.05
75	October	20.12
76	October	14.8
77	October	16.88
78	October	18.07
79	October	13.8
80	October	18.49
81	October	17.24
82	October	24.78
83	October	16.11
84	October	13.41
85	October	20.62
86	October	16.63

Data set of CO_2 efflux rates of burrows (F_b)

Some burrows or measurments did not yield a value due to technical difficulties.

Crab burrow	Time	Measurment	F_b (µmol m^{-2} s^{-1})
1	April	1	7.0084937
1	April	2	5.6428533
1	April	3	6.0061026
1	April	4	5.7149996
1	April	5	6.0426175
2	April	1	3.9364265
2	April	2	3.798566
2	April	3	3.823926
2	April	4	3.8476071
2	April	5	3.7928506
3	April	1	7.1028333
3	April	2	4.3648481
3	April	3	3.8084219
3	April	4	3.6478559
3	April	5	4.8317249
4	April	1	4.6700758
4	April	2	3.9146601
4	April	3	3.6834689
4	April	4	3.3753889
4	April	5	3.4210035
5	April	1	16.5310568
5	April	2	11.8720165
5	April	3	8.1079703
5	April	4	7.8082039
5	April	5	8.4852839
6	April	1	1.2356676
6	April	2	1.2809451
6	April	3	1.2298262
6	April	4	1.2693422
7	April	1	5.1555658
7	April	2	6.3735045
7	April	3	5.6000888
7	April	4	7.6902971
7	April	5	6.2494824
8	April	1	4.9603861
8	April	2	5.2241666
8	April	3	4.7416246
8	April	4	5.7472462
8	April	5	5.2251445
9	April	1	3.5749822
9	April	2	3.555753
9	April	3	8.122535
9	April	4	6.8358688
9	April	5	12.8109185
10	April	1	17.3017181
10	April	2	12.4427502
10	April	3	14.6416235
10	April	4	14.440647

10	April	5	12.4029061
11	April	1	12.5453857
11	April	2	6.0557769
11	April	3	5.6074497
11	April	4	5.0129583
11	April	5	8.238401
12	April	1	4.2453905
12	April	2	3.7833649
12	April	3	3.7277011
12	April	4	3.3911701
12	April	5	3.3032109
13	April	1	21.7928085
13	April	2	15.9614882
13	April	3	12.3640646
13	April	4	11.4571324
13	April	5	5.6470903
14	April	1	12.4834138
14	April	2	8.3188061
14	April	3	8.7872085
14	April	4	8.8212808
14	April	5	7.7326137
15	April	1	6.2425457
15	April	2	5.3558445
15	April	3	4.8268969
15	April	4	5.6812491
15	April	5	5.9761293
16	April	1	5.9588629
16	April	2	5.1417803
16	April	3	5.0765762
16	April	4	5.9391077
16	April	5	7.3855009
17	April	1	13.3930358
17	April	2	7.1586053
17	April	3	10.3159851
17	April	4	8.5797187
17	April	5	5.5790319
18	April	1	16.2705249
18	April	2	13.6091419
18	April	3	18.0655279
18	April	4	12.4821757
19	April	1	11.4462942
19	April	2	6.2625821
19	April	3	7.5772165
19	April	4	7.1015004
19	April	5	5.9171708
20	April	1	8.9700696
20	April	3	22.8201743
20	April	4	15.1988954
20	April	5	8.7488757
21	April	1	5.6759875
21	April	2	4.8704224
21	April	3	6.4586139

21	April	4	4.207868
21	April	5	4.8705566
22	April	1	2.081937
22	April	2	2.1455084
22	April	3	2.2497082
22	April	4	2.0885097
22	April	5	2.1055682
23	April	1	4.3676557
23	April	2	4.2381849
23	April	3	4.4608288
23	April	4	4.621497
23	April	5	4.3139194
24	April	1	12.6714791
24	April	2	8.7415877
24	April	3	6.4904542
24	April	4	6.2085183
31	July	1	3.5696977
31	July	2	3.8101643
31	July	3	3.9753309
31	July	4	3.6263326
31	July	5	5.0679486
32	July	1	3.4954726
32	July	2	3.327553
32	July	3	2.8990743
32	July	4	3.5628774
32	July	5	3.3323276
33	July	1	2.065765
33	July	2	2.0034049
33	July	3	2.4116405
33	July	4	2.6948141
33	July	5	2.9032352
34	July	1	3.6263601
34	July	2	2.493836
34	July	3	2.2374192
34	July	4	2.3499559
34	July	5	3.2301003
35	July	1	2.6541471
35	July	2	2.1592119
35	July	3	1.9295006
35	July	4	1.732503
35	July	5	2.3491852
36	July	1	12.941279
36	July	2	9.7955249
36	July	3	13.6200276
36	July	4	9.9558967
36	July	5	7.6667279
37	July	1	4.3252719
37	July	2	4.2274051
37	July	3	3.784737
37	July	4	4.7542489
37	July	5	7.1800688
38	July	1	6.4278592

38	July	2	6.8889414
38	July	3	5.5711086
38	July	4	3.9486678
38	July	5	5.2466302
39	July	1	1.7421829
39	July	2	2.424683
39	July	3	1.959019
39	July	4	2.5217678
39	July	5	2.1274259
40	July	1	4.281536
40	July	2	7.5483667
40	July	3	7.0132038
40	July	4	3.554771
40	July	5	6.0497054
41	July	1	7.1575819
41	July	2	8.3445894
41	July	3	7.7859129
41	July	4	7.5467455
41	July	5	7.4041741
42	July	1	2.4611495
42	July	2	2.61807
42	July	3	2.3443149
42	July	4	2.480643
42	July	5	2.0866537
43	July	1	3.7188617
43	July	2	5.7655617
43	July	3	4.7496716
43	July	4	3.868029
43	July	5	3.6447265
44	July	1	7.1499321
44	July	2	6.4563293
44	July	3	8.0369799
44	July	4	8.3372346
44	July	5	6.7076589
45	July	1	18.080208
45	July	2	12.6141666
45	July	3	12.2811517
45	July	4	9.8340701
45	July	5	8.9337729
46	July	1	1.8396279
46	July	2	2.4161107
46	July	3	2.6225395
46	July	4	4.1515543
46	July	5	1.7876338
47	July	1	1.5362443
47	July	2	1.4491783
47	July	3	1.0922951
47	July	4	1.5449702
47	July	5	1.7521747
48	July	1	14.5584716
48	July	2	13.3508653
48	July	3	11.4445574

48	July	4	11.6656969
48	July	5	11.3420101
49	July	1	41.5843997
49	July	2	34.8142833
49	July	3	31.621091
49	July	4	31.2780751
49	July	5	29.3090873
50	July	1	15.0148372
50	July	2	12.5467918
50	July	3	11.0222378
50	July	4	16.2393282
50	July	5	13.5139257
51	July	1	32.5869527
51	July	2	28.1993525
51	July	3	27.8572322
51	July	4	24.092388
51	July	5	22.3120242
52	July	1	14.4071475
52	July	2	8.5266109
52	July	3	8.3965641
52	July	4	14.0460223
52	July	5	9.5239026
53	July	1	35.7766827
53	July	2	36.1471689
53	July	3	33.3692798
53	July	4	38.46252
53	July	5	37.742393
54	July	2	40.1682508
54	July	3	37.7842827
54	July	4	33.8858889
54	July	5	34.2510152
55	September	1	2.6798773
55	September	2	1.1398294
55	September	3	0.9059706
55	September	4	1.2324487
55	September	5	1.138443
56	September	1	3.1880116
56	September	2	3.1870945
56	September	3	3.128654
56	September	4	3.1977758
56	September	5	3.3307884
57	September	1	3.726173
57	September	2	7.1444949
57	September	3	2.9173161
57	September	4	3.8683356
57	September	5	4.2767747
58	September	1	11.8377672
58	September	2	9.1618616
58	September	3	12.2781286
58	September	4	12.9841886
58	September	5	11.3767585
59	September	1	3.244799

59	September	2	3.4301614
59	September	3	3.4330299
59	September	4	3.6046944
59	September	5	3.0186076
60	September	1	5.2933536
60	September	2	3.9404225
60	September	3	4.7019616
60	September	4	4.5865632
60	September	5	5.3769336
61	September	1	1.9801158
61	September	2	2.1912318
61	September	3	2.2838846
61	September	4	2.6585388
61	September	5	2.440157
62	September	1	4.612161
62	September	2	6.0742573
62	September	3	3.6716009
62	September	4	4.477764
62	September	5	4.1825886
63	September	1	14.2655041
63	September	2	6.4905757
63	September	3	4.616546
63	September	4	4.1244067
63	September	5	6.8646528
64	September	1	8.3287744
64	September	2	8.3154796
64	September	3	8.6431362
64	September	4	6.8179552
64	September	5	7.0228286
65	September	1	6.8107395
65	September	2	7.5131295
65	September	3	8.0544905
65	September	4	7.2280907
65	September	5	7.9474049
66	September	1	6.8000681
66	September	2	7.3429933
66	September	3	7.898635
66	September	4	6.813846
66	September	5	6.5760949
67	September	1	3.4999919
67	September	2	2.8146017
67	September	3	3.3490975
67	September	4	5.5002678
67	September	5	3.8689795
69	September	1	7.9934381
69	September	2	8.918983
69	September	3	12.0308275
69	September	4	8.6673564
69	September	5	9.2564161
70	September	1	5.1282215
70	September	2	5.2063334
70	September	3	6.2767877

70	September	4	5.3320878
70	September	5	5.8268407
71	September	1	6.5350116
71	September	2	6.0100062
71	September	3	5.3676842
71	September	4	6.2700581
71	September	5	8.3366683
72	September	1	5.306254
72	September	2	5.5158915
72	September	3	5.6975925
72	September	4	5.3098439
72	September	5	5.8645286
73	September	1	3.4660631
73	September	2	3.1807906
73	September	3	3.1337351
73	September	4	3.7285413
73	September	5	3.6259786
74	September	1	3.55941
74	September	2	17.8639568
74	September	3	11.3921431
74	September	4	9.680552
74	September	5	11.2160779
75	October	1	6.7331339
75	October	2	5.3073742
75	October	3	8.1232182
75	October	4	5.9482654
75	October	5	5.6234843
76	October	1	8.8659164
76	October	2	8.6455065
76	October	3	7.5705047
76	October	4	7.5427118
76	October	5	7.2598173
77	October	1	9.4962302
77	October	2	7.6302471
77	October	3	5.2035874
77	October	4	5.5618363
77	October	5	5.4561809
78	October	1	9.3486774
78	October	2	9.0515461
78	October	3	8.5439095
78	October	4	11.9804566
78	October	5	10.581361
79	October	1	5.017106
79	October	2	4.9525669
79	October	3	4.9057641
79	October	4	4.9342853
79	October	5	5.8170833
80	October	1	7.5516971
80	October	2	5.6571682
80	October	3	6.1277598
80	October	4	6.2843863
80	October	5	7.2035084

81	October	1	2.5346882
81	October	2	2.2601188
81	October	3	3.2509097
81	October	4	3.5705355
81	October	5	6.7745144
82	October	1	1.3685963
82	October	2	1.5330542
82	October	3	3.1577839
82	October	4	1.6613109
82	October	5	2.1551276
83	October	1	4.2007062
83	October	2	3.8302672
83	October	3	3.6940417
83	October	4	5.315129
83	October	5	5.2474687
84	October	1	16.264476
84	October	2	12.6589088
84	October	3	12.0441423
84	October	4	13.7491943
84	October	5	12.6300833
86	October	1	3.5697742
86	October	2	4.9625191
86	October	3	3.1891278
86	October	4	3.4304562
86	October	5	1.5773547

Data set of control CO_2 efflux rates (plain sediment)

In April one sampling site could not be measured.
Occasionally measurement did not yield any result,
because of technical difficulties.

Time	Sampling point	Measurement	CO_2 efflux rate of plain sediment (µmol m^{-2} s^{-1})
April	1	1	1.13921358
April	1	2	1.112771787
April	1	3	1.202517687
April	1	4	1.213407417
April	2	1	0.593992756
April	2	2	0.621491792
April	2	3	0.653988079
April	2	4	0.670763746
April	3	1	0.471732539
April	3	2	0.609743484
April	3	3	0.685344007
April	3	4	0.746645483
April	4	1	0.639523581
April	4	2	0.741012817
April	4	3	0.806009118
April	4	4	0.845608027
April	5	1	1.184742685
April	5	2	1.161585437
April	5	3	1.165557754
April	5	4	1.138900795
April	6	1	0.712363214
April	6	2	0.642647777
April	6	3	0.730958316
April	6	4	0.712225022
April	7	1	0.913457543
April	7	2	0.470923383
April	7	3	0.462110261
April	7	4	0.489674605

April	8	1	0.593724573
April	8	2	0.608809695
April	8	3	0.609395043
April	8	4	0.639633416
April	9	1	0.491383252
April	9	2	0.62351525
April	9	3	0.498762223
April	9	4	0.463324437
April	10	1	0.568137494
April	10	2	0.53879773
April	10	3	0.443102696
April	10	4	0.563017637
April	11	1	0.455824015
April	11	2	0.491976295
April	11	3	0.488819309
April	11	4	0.476207222
April	12	1	0.612804444
April	12	2	0.604180099
April	12	3	0.749455157
April	12	4	0.926226683
April	13	1	0.298738269
April	13	2	0.310957841
April	13	3	0.309338762
April	13	4	0.325153937
April	14	1	2.922090383
April	14	2	2.890783693
April	14	3	3.074609574
April	14	4	3.081545307
April	15	1	0.467763142
April	15	2	0.478020027
April	15	3	0.389021439
April	15	4	0.362235008
April	16	1	0.618294412
April	16	2	0.644675673

April	16	3	0.506672382
April	16	4	0.546956744
April	17	1	0.597168108
April	17	2	0.663555678
April	17	3	0.622166058
April	17	4	0.629392556
April	19	1	1.975411919
April	19	2	1.2354959
April	19	3	1.197056909
April	19	4	1.093025009
April	20	1	0.583994737
April	20	2	0.626962341
April	20	3	2.43802485
April	20	4	0.239367996
April	21	1	0.031221509
April	22	1	0.030525008
April	22	2	0.12504295
April	22	3	0.225399934
April	22	4	0.324817046
April	23	1	0.009045701
April	23	2	0.02349414
April	23	3	0.032714845
April	24	1	0.080316299
April	24	2	0.088964703
April	24	3	0.138717046
April	24	4	0.138926654
July	25	1	2.250957343
July	25	2	1.024235134
July	25	3	0.975886784
July	25	4	1.018724086
July	26	1	1.078439764
July	26	2	1.03792679
July	26	3	1.074935955
July	26	4	1.006281781

July	27	1	0.479108934
July	27	2	0.496119108
July	27	3	0.253337481
July	27	4	0.527184147
July	28	1	0.679198692
July	28	2	0.807476548
July	28	3	0.798323316
July	28	4	0.872539225
July	29	1	0.774008755
July	29	2	0.775924599
July	29	3	0.878558541
July	29	4	0.822419521
July	30	1	0.629844579
July	30	2	0.745957548
July	30	3	0.699241569
July	30	4	0.71954819
July	31	1	1.52050605
July	31	2	0.569311163
July	31	3	0.562199893
July	31	4	0.573747976
July	32	1	0.361743664
July	32	2	0.383240936
July	32	3	0.412284825
July	32	4	0.390634875
July	33	1	0.938900631
July	33	2	1.56828953
July	33	3	1.027940962
July	33	4	0.858347489
July	34	1	0.599671695
July	34	2	0.565558566
July	34	3	0.543518864
July	34	4	0.531503424
July	35	1	0.595701838
July	35	2	0.586234582

July	35	3	0.58374112
July	35	4	0.527842984
July	36	1	0.864089818
July	36	2	0.808844412
July	36	3	0.856974397
July	36	4	0.914499352
July	37	1	1.46126477
July	37	2	0.618735011
July	37	3	0.838555113
July	37	4	0.827287104
July	38	1	0.87579619
July	38	2	0.849748502
July	38	3	0.887192132
July	38	4	0.864593712
July	39	1	0.186030891
July	39	2	0.051042797
July	39	3	0.292015031
July	39	4	0.213403252
July	40	1	0.53788951
July	40	2	0.416696371
July	40	3	0.623437755
July	40	4	0.688511321
July	41	1	5.478321327
July	41	2	1.158714071
July	41	3	1.184779903
July	41	4	1.184015608
July	42	1	0.51088379
July	42	2	0.532002771
July	42	3	0.53089586
July	42	4	3.83066347
July	43	1	1.207115418
July	43	2	0.884991622
July	43	3	0.864835843
July	43	4	0.897819013

July	44	1	1.001249661
July	44	2	0.962890276
July	44	3	0.993606899
July	44	4	0.946103314
July	45	1	0.78077457
July	45	2	0.7222328
July	45	3	0.741106095
July	45	4	0.691730904
July	46	1	0.78624004
July	46	2	0.808730803
July	46	3	0.765208842
July	46	4	0.778234966
July	47	1	0.756502534
July	47	2	0.782183951
July	47	3	0.799519112
July	47	4	0.787042512
July	48	1	0.866626442
July	48	2	0.952490455
July	48	3	0.835053931
July	48	4	0.870204389
September	49	1	0.539651604
September	49	2	0.779206505
September	49	3	1.007509745
September	49	4	0.732552744
September	50	1	1.122606618
September	50	2	0.991031151
September	50	3	1.125291236
September	50	4	1.010625577
September	51	1	0.937239244
September	51	2	0.354225754
September	51	3	0.231020225
September	52	1	1.3971204
September	52	2	1.624571666
September	52	3	0.849395523

September	52	4	0.586899661
September	53	1	1.406506687
September	53	2	1.409955858
September	53	3	1.37396037
September	53	4	1.525239398
September	54	1	1.408278397
September	54	2	1.593355271
September	54	3	1.633182335
September	54	4	1.80057132
September	55	1	0.229994051
September	55	2	0.269937815
September	55	3	0.347560545
September	56	1	0.355398474
September	56	2	0.56870767
September	56	3	0.116774007
September	56	4	0.163755159
September	57	1	0.418373692
September	57	2	1.062227964
September	57	3	0.385810322
September	57	4	0.392436901
September	58	1	0.959340263
September	58	2	1.023018436
September	58	3	0.93310565
September	58	4	0.921540711
September	59	1	0.973635885
September	59	2	0.969591736
September	59	3	1.106264534
September	59	4	1.046622285
September	60	1	1.147282563
September	60	2	1.104388469
September	60	3	1.113020841
September	60	4	1.100770363
September	61	1	2.235884636
September	61	2	1.527803319

September	61	3	2.474480615
September	61	4	1.888890718
September	62	1	1.211801454
September	62	2	1.154717038
September	62	3	1.373174606
September	62	4	1.034719821
September	63	1	1.905736279
September	63	2	0.993041977
September	63	3	1.101025052
September	63	4	1.131274287
September	64	1	0.570909492
September	64	2	0.559593238
September	64	3	0.736208108
September	64	4	0.605147487
September	65	1	1.450286414
September	65	2	1.600522767
September	65	3	1.655827417
September	65	4	1.177762983
September	66	1	1.268249508
September	66	2	1.771304027
September	66	3	1.74060441
September	66	4	1.517465818
September	67	1	2.623251372
September	67	2	2.760824151
September	67	3	2.858667522
September	67	4	2.923919851
September	68	1	2.620926361
September	68	2	2.792783811
September	68	3	2.555237562
September	68	4	2.687153368
September	69	1	1.404471055
September	69	2	1.466347325
September	69	3	1.497468577
September	69	4	1.447689599

September	70	1	2.073924877
September	70	2	2.42258067
September	70	3	2.20912527
September	70	4	1.999183648
September	71	1	1.904210058
September	71	2	2.120968417
September	71	3	1.926354172
September	71	4	2.010888576
September	72	1	1.903533024
September	72	2	2.384620288
September	72	3	2.026853806
October	73	1	2.893083237
October	73	2	1.382008554
October	73	3	1.25361488
October	73	4	1.444981598
October	74	1	0.54077041
October	74	2	0.765704728
October	74	3	0.824776612
October	74	4	0.836621484
October	75	1	0.89274656
October	75	2	0.981642057
October	75	3	1.052115119
October	75	4	1.053533611
October	76	1	0.506695484
October	76	2	0.68445091
October	76	3	0.749826926
October	76	4	0.778118408
October	77	1	0.801134749
October	77	2	0.854315816
October	77	3	0.873132882
October	77	4	0.789909682
October	78	1	1.031232136
October	78	2	1.067808177
October	78	3	1.12930678

October	78	4	1.135551894
October	79	1	1.11799543
October	79	2	1.119626454
October	79	3	1.11323639
October	79	4	1.097085341
October	80	1	1.310523042
October	80	2	1.268184468
October	80	3	1.293269004
October	80	4	1.288591922
October	81	1	0.92810195
October	81	2	0.978776428
October	81	3	0.976136022
October	81	4	1.053446569
October	82	1	1.161763799
October	82	2	1.371248665
October	82	3	1.283705154
October	82	4	1.857834289
October	83	1	0.97150384
October	83	2	1.090089435
October	83	3	1.286009222
October	83	4	1.235247288
October	84	1	1.552814427
October	84	2	1.479455214
October	84	3	0.931872893
October	84	4	0.923117981
October	85	1	2.906159387
October	85	2	1.485181194
October	85	3	1.323137754
October	85	4	1.431612617
October	86	1	1.049159896
October	86	2	1.054824908
October	86	3	1.114349953
October	86	4	1.30398149
October	87	1	1.550168907

October	87	2	1.483231256
October	87	3	1.479187307
October	87	4	1.525829402
October	88	1	0.417253038
October	88	2	0.630109816
October	88	3	0.580099459
October	88	4	0.687147412
October	89	1	4.028267708
October	89	2	2.612737695
October	89	3	3.503666281
October	89	4	3.281453704
October	90	1	1.570045722
October	90	2	1.562771914
October	90	3	1.628032813
October	90	4	1.701648598
October	91	1	2.234640057
October	91	2	1.042483752
October	91	3	1.026553381
October	91	4	1.198201798
October	92	1	1.074743509
October	92	2	1.287615717
October	92	3	1.332320327
October	92	4	1.361028246
October	93	1	1.475802434
October	93	2	1.449795525
October	93	3	1.424375131
October	93	4	1.386220804
October	94	1	1.255124553
October	94	2	1.262417563
October	94	3	1.38803761
October	94	4	1.415373032
October	95	1	0.916487
October	95	2	0.99362653
October	95	3	1.020702475

October	95	4	1.050836352
October	96	1	0.384791499
October	96	2	0.779293534
October	96	3	0.807596563
October	96	4	1.018775275

Data set of carapace widths of *Ucides cordatus*

Crab	Carapace width (cm)	Treatment
1	7	in air
2	7.1	in air
3	6.9	in air
4	7.4	in air
5	7.3	in air
6	7.4	in air
7	7.2	in air
8	7.6	in air
9	7.8	in air
10	7.8	in air
11	7.7	in air
12	7.6	in air
13	7.2	in air
14	6.5	in air
15	7.3	in sea water
16	7.5	in sea water
17	6.7	in sea water
18	7.1	in sea water
19	6.7	in sea water
20	6.9	in sea water
21	6.9	in sea water
22	6.8	in sea water
23	7.4	in sea water
24	7	in sea water
25	7.3	in sea water
26	6.2	in sea water
27	7.4	in sea water
28	6.5	in sea water

Data set of crab respiration

Measurement	Crab	Treatment	Crab respiration (µmol CO_2 kg^{-1} s^{-1})
1	1	in air	0.772217306
2	1	in air	0.544718748
3	1	in air	0.846268802
1	2	in air	0.873972546
2	2	in air	0.816937423
1	3	in air	0.872620594
2	3	in air	0.426101252
3	3	in air	1.010319889
4	3	in air	0.674458136
5	3	in air	0.512294575
1	4	in air	3.94324705
2	4	in air	2.062113485
3	4	in air	1.183894614
4	4	in air	1.335128757
5	4	in air	1.439283789
1	5	in air	2.889967275
2	5	in air	1.417610336
3	5	in air	0.915343711
4	5	in air	0.856326727
5	5	in air	0.95281562
1	6	in air	2.034565796
2	6	in air	1.24090179
3	6	in air	0.922850555
4	6	in air	0.615492352
5	6	in air	0.717326825
1	7	in air	1.646410476
2	7	in air	0.925418392
3	7	in air	0.830916778
4	7	in air	0.758456092
5	7	in air	0.630308958
1	8	in air	2.466847467
2	8	in air	1.576370113
3	8	in air	0.987515571
4	8	in air	0.940783296
5	8	in air	0.741828424
1	9	in air	3.501228214
2	9	in air	2.140982215
3	9	in air	1.683005133
4	9	in air	1.179200655
5	9	in air	1.119255607
1	10	in air	1.922006316
2	10	in air	1.390110687
3	10	in air	1.213053993
4	10	in air	0.976777086
5	10	in air	0.705905603
1	11	in air	0.781751673
2	11	in air	0.57391499
3	11	in air	0.375763327
4	11	in air	0.421521187

5	11	in air	0.497671074
3	12	in air	1.272754285
4	12	in air	0.589584019
5	12	in air	0.833750761
1	13	in air	3.765084631
2	13	in air	2.800651846
3	13	in air	1.820897057
4	13	in air	2.036065457
5	13	in air	1.502376128
1	14	in air	4.079028619
2	14	in air	1.713180635
3	14	in air	0.980646903
4	14	in air	0.689960439
5	14	in air	0.826407984
1	15	in sea water	1.90458296
2	15	in sea water	1.959375624
3	15	in sea water	1.654414318
4	15	in sea water	1.615881489
5	15	in sea water	1.321830338
1	16	in sea water	1.731410697
1	17	in sea water	1.895090312
2	17	in sea water	1.406163159
3	17	in sea water	1.253432871
4	17	in sea water	0.426064369
5	17	in sea water	0.29833501
1	18	in sea water	2.391441191
2	18	in sea water	1.49366973
3	18	in sea water	0.44200516
4	18	in sea water	0.485045354
5	18	in sea water	0.63813249
1	19	in sea water	0.836075526
2	19	in sea water	0.115952116
3	19	in sea water	0.163512415
4	19	in sea water	0.365498125
5	19	in sea water	0.898565475
1	20	in sea water	1.289199725
2	20	in sea water	0.135954079
3	20	in sea water	0.865425267
4	20	in sea water	0.382119335
5	20	in sea water	0.040062837
1	21	in sea water	0.383276895
2	21	in sea water	0.308988284
3	21	in sea water	0.449213623
4	21	in sea water	0.489749184
5	21	in sea water	0.692793589
1	22	in sea water	1.810319458
2	22	in sea water	1.493937641
3	22	in sea water	1.60622981
4	22	in sea water	1.574589014
5	22	in sea water	1.944136963
1	23	in sea water	2.375689832
2	23	in sea water	2.180110224

3	23	in sea water	1.804620035
4	23	in sea water	1.525574493
5	23	in sea water	1.287262821
1	24	in sea water	1.217563644
1	25	in sea water	1.075643384
2	25	in sea water	1.126509184
3	25	in sea water	0.62669035
4	25	in sea water	0.802019998
5	25	in sea water	0.658585977
1	26	in sea water	0.779542369
2	26	in sea water	0.623571321
3	26	in sea water	0.497104746
4	26	in sea water	0.466641634
5	26	in sea water	0.406007891
2	27	in sea water	0.835470905
3	27	in sea water	0.652566253
4	27	in sea water	0.503141202
5	27	in sea water	0.467261895
1	28	in sea water	0.604667506
2	28	in sea water	0.732538432
3	28	in sea water	0.597271497
4	28	in sea water	0.340628483
5	28	in sea water	0.499979593

Data set of rH values in the burrow wall sediment (BWS)

Some of the burrows or sediment depths could not be measured due to technical difficulties.

Crab burrow	Time	Sediment depth (cm)	Horizontal distance (cm)	rH
1	April	1	2	26.7
1	April	1	5	16.93
1	April	1	8	19.37
1	April	1	15	17.66
1	April	10	2	15.81
1	April	10	5	15.12
1	April	10	8	15.67
1	April	10	15	17.96
1	April	30	2	14.16
1	April	30	5	13.56
1	April	30	8	9.16
1	April	30	15	11.99
1	April	50	2	9.65
1	April	50	5	9.23
1	April	50	8	8.76
1	April	50	15	9.79
2	April	1	2	10.91
2	April	1	5	10.4
2	April	1	8	10.83
2	April	1	15	14.13
2	April	10	2	9.29
2	April	10	5	8.62
2	April	10	8	8.23
2	April	10	15	7.13
2	April	30	2	7.32
2	April	30	5	7.08
2	April	30	8	6.91
2	April	30	15	7.52
2	April	50	2	7.57

2	April	50	5	6.89
2	April	50	8	6.64
2	April	50	15	7.19
3	April	1	2	11.94
3	April	1	5	10.73
3	April	1	8	14.66
3	April	1	15	13.04
3	April	10	2	7.59
3	April	10	5	7.17
3	April	10	8	6.95
3	April	10	15	7.71
3	April	30	2	8.09
3	April	30	5	7.8
3	April	30	8	7.58
3	April	30	15	7.6
3	April	50	2	7.79
3	April	50	5	7.57
3	April	50	8	7.28
3	April	50	15	7.02
4	April	1	2	13.38
4	April	1	5	11.54
4	April	1	8	10.9
4	April	1	15	12.67
4	April	10	2	8.83
4	April	10	5	7.78
4	April	10	8	7.17
4	April	10	15	8.03
4	April	30	2	8.07
4	April	30	5	7.74
4	April	30	8	7.51
4	April	30	15	8.14
5	April	1	2	19.16
5	April	1	5	16.03

5	April	1	8	15.5
5	April	1	15	17.72
5	April	10	2	11.61
5	April	10	5	10.61
5	April	10	8	10.37
5	April	10	15	11.1
5	April	30	2	7.76
5	April	30	5	7.28
5	April	30	8	7.25
5	April	30	15	7.81
5	April	50	2	8.14
5	April	50	5	7.9
5	April	50	8	7.76
5	April	50	15	7.5
6	April	1	2	13.74
6	April	1	5	13.65
6	April	1	8	18.51
6	April	1	15	11.74
6	April	10	2	8.31
6	April	10	5	8.04
6	April	10	8	7.79
6	April	10	15	7.79
6	April	30	2	7.78
6	April	30	5	7.54
6	April	30	8	7.4
6	April	30	15	7.61
6	April	50	2	8.73
6	April	50	5	8.05
6	April	50	8	7.83
6	April	50	15	7.77
7	April	1	2	17.93
7	April	1	5	17.98
7	April	1	8	19.95

7	April	1	15	21.24
8	April	1	2	9.7
8	April	1	5	10.1
8	April	1	8	16.7
8	April	1	15	15.32
8	April	10	2	16.66
8	April	10	5	15.96
8	April	10	8	15.24
8	April	10	15	16.17
8	April	30	2	8.87
8	April	30	5	8.08
8	April	30	8	9.94
8	April	30	15	8.82
8	April	50	2	9.51
8	April	50	5	8.85
8	April	50	8	7.64
8	April	50	15	7.91
9	April	1	2	15.98
9	April	1	5	19.08
9	April	1	8	16.36
9	April	1	15	14.28
9	April	10	2	16.68
9	April	10	5	16.23
9	April	10	8	15.06
9	April	10	15	12.02
9	April	30	2	13.32
9	April	30	5	12.18
9	April	30	8	13.74
9	April	30	15	12.03
9	April	50	2	9.52
9	April	50	5	8.94
9	April	50	8	8.59
9	April	50	15	9.11

13	April	1	2	10.65
13	April	1	5	11.29
13	April	1	8	10.73
13	April	1	15	11.31
13	April	10	2	15
13	April	10	5	13.27
13	April	10	8	12.66
13	April	10	15	12.16
13	April	30	2	13.12
13	April	30	5	10.66
13	April	30	8	10.22
13	April	30	15	9.49
14	April	1	2	14.09
14	April	1	5	15.11
14	April	1	8	14.07
14	April	1	15	15.95
14	April	10	2	12.2
14	April	10	5	12.07
14	April	10	8	11.66
14	April	10	15	13.13
14	April	30	2	15.29
14	April	30	5	15.24
14	April	30	8	14.93
14	April	30	15	14.03
15	April	1	2	15.14
15	April	1	5	14.62
15	April	1	8	13.18
15	April	1	15	18.29
15	April	10	2	15.44
15	April	10	5	15.38
15	April	10	8	15.05
15	April	10	15	11.97
15	April	30	2	13

15	April	30	5	11.68
15	April	30	8	11.42
15	April	30	15	9.69
16	April	1	2	18.27
16	April	1	5	18.49
16	April	1	8	18.15
16	April	1	15	18.52
16	April	10	2	14.36
16	April	10	5	14.37
16	April	10	8	14.1
16	April	10	15	12.6
16	April	30	2	11.23
16	April	30	5	11.47
16	April	30	8	11.17
16	April	30	15	11.54
16	April	50	2	11.55
16	April	50	5	11.36
16	April	50	8	9.74
16	April	50	15	9.76
17	April	1	2	13.99
17	April	1	5	12.93
17	April	1	8	13.73
17	April	1	15	14.54
17	April	10	2	15.44
17	April	10	5	15.42
17	April	10	8	14.57
17	April	10	15	13.56
17	April	30	2	12.71
17	April	30	5	13.18
17	April	30	8	12.88
17	April	30	15	12.36
17	April	50	2	13.39
17	April	50	5	12.97

17	April	50	8	12.65
17	April	50	15	12.73
18	April	1	2	13.11
18	April	1	5	12.29
18	April	1	8	13.81
18	April	1	15	14.53
18	April	10	2	14.4
18	April	10	5	13.04
18	April	10	8	13.54
18	April	10	15	15.76
19	April	1	2	11.49
19	April	1	5	11.84
19	April	1	8	12.45
19	April	1	15	13.14
19	April	10	2	12.38
19	April	10	5	11.04
19	April	10	8	11.15
19	April	10	15	16.08
19	April	30	2	8.67
19	April	30	5	8.52
19	April	30	8	8.37
19	April	30	15	7.92
20	April	1	2	14.59
20	April	1	5	14.14
20	April	1	8	14.13
20	April	1	15	15.5
20	April	10	2	10.94
20	April	10	5	11.03
20	April	10	8	10.63
20	April	10	15	12.03
20	April	30	2	8.45
20	April	30	5	8
20	April	30	8	8.13

20	April	30	15	9.12
21	April	1	2	15.25
21	April	1	5	15.5
21	April	1	8	14.97
21	April	1	15	13.64
21	April	10	2	16.81
21	April	10	5	16.15
21	April	10	8	15.04
21	April	10	15	15.06
21	April	30	2	12.99
21	April	30	5	12.18
21	April	30	8	11.67
21	April	30	15	13.57
21	April	50	2	10.35
21	April	50	5	10.37
21	April	50	8	9.62
21	April	50	15	10.25
22	April	1	2	16.07
22	April	1	5	15.36
22	April	1	8	13.01
22	April	1	15	12.56
22	April	10	2	13.62
22	April	10	5	12.75
22	April	10	8	11.94
22	April	10	15	14.07
22	April	30	2	13.62
22	April	30	5	12.88
22	April	30	8	12.42
22	April	30	15	12.61
22	April	50	2	10.84
22	April	50	5	10.18
22	April	50	8	9.74
22	April	50	15	9.86

31	July	1	2	16.86
31	July	1	5	15.33
31	July	1	8	15.8
31	July	1	15	15.67
31	July	10	2	20.39
31	July	10	5	18.04
31	July	10	8	16.82
31	July	10	15	20.49
31	July	30	2	17.08
31	July	30	5	13.87
31	July	30	8	13.65
31	July	30	15	13.01
32	July	1	2	16.76
32	July	1	5	15.97
32	July	1	8	16.17
32	July	1	15	14.83
32	July	10	2	12.23
32	July	10	5	11.78
32	July	10	8	12.06
32	July	10	15	11.53
33	July	1	2	14.01
33	July	1	5	12.88
33	July	1	8	16.09
33	July	1	15	15.96
33	July	10	2	12.06
33	July	10	5	12.24
33	July	10	8	12.34
33	July	10	15	13.79
33	July	30	2	10.87
33	July	30	5	11.11
33	July	30	8	10.48
33	July	30	15	11.37
34	July	1	2	14.25

34	July	1	5	14.21
34	July	1	8	12.13
34	July	1	15	12.27
34	July	10	2	9.41
34	July	10	5	9.33
34	July	10	8	8.72
34	July	10	15	14.51
34	July	30	2	11.54
34	July	30	5	11.39
34	July	30	8	11.33
34	July	30	15	11.39
35	July	1	2	14.07
35	July	1	5	15.17
35	July	1	8	14.95
35	July	1	15	16.21
35	July	10	2	15.85
35	July	10	5	15.71
35	July	10	8	14.05
35	July	10	15	15.17
35	July	30	2	16.33
35	July	30	5	15.67
35	July	30	8	15.28
35	July	30	15	15.24
36	July	1	2	14.6
36	July	1	5	13.86
36	July	1	8	16.49
36	July	1	15	16.89
36	July	10	2	12.7
36	July	10	5	10.83
36	July	10	8	11.4
36	July	10	15	14.28
36	July	30	2	11.19
36	July	30	5	11.23

36	July	30	8	11.62
36	July	30	15	12.39
37	July	1	2	17.13
37	July	1	5	19.05
37	July	1	8	14.16
37	July	1	15	13.89
37	July	10	2	14.79
37	July	10	5	14.72
37	July	10	8	14.71
37	July	10	15	13.67
37	July	30	2	15.36
37	July	30	5	15.5
37	July	30	8	15.22
38	July	1	2	17.57
38	July	1	5	16.91
38	July	1	8	16.22
38	July	1	15	17.22
38	July	10	2	16.81
38	July	10	5	16.53
38	July	10	8	16.42
38	July	10	15	15.82
38	July	30	2	15.77
38	July	30	5	15.1
38	July	30	8	14.55
38	July	30	15	12.53
39	July	1	2	26.03
39	July	1	5	26.42
39	July	1	8	24.56
39	July	1	15	20.29
39	July	10	2	14.53
39	July	10	5	14.13
39	July	10	8	13.84
39	July	10	15	15.79

39	July	30	2	12.1
39	July	30	5	10.79
39	July	30	8	10.82
39	July	30	15	8.92
40	July	1	2	18.31
40	July	1	5	17.21
40	July	1	8	16.14
40	July	1	15	20.44
40	July	10	2	16.08
40	July	10	5	15.58
40	July	10	8	15.11
40	July	10	15	17.44
40	July	30	2	13.3
40	July	30	5	12.59
40	July	30	8	11.71
40	July	30	15	12.22
41	July	1	2	16.39
41	July	1	5	15.69
41	July	1	8	15.61
41	July	1	15	15.93
41	July	10	2	15.22
41	July	10	5	15.09
41	July	10	8	14.64
41	July	10	15	13.57
41	July	30	2	10.82
41	July	30	5	10.29
41	July	30	8	10.41
41	July	30	15	10.47
41	July	50	2	12.12
41	July	50	5	13.09
41	July	50	8	12.82
41	July	50	15	9.02
42	July	1	2	16.66

42	July	1	5	14.66
42	July	1	8	17.04
42	July	1	15	17.48
42	July	10	2	14.43
42	July	10	5	14.43
42	July	10	8	13.95
42	July	10	15	14.28
42	July	30	2	11.25
42	July	30	5	10.07
42	July	30	8	9.28
42	July	30	15	8.09
42	July	50	2	9.63
42	July	50	5	9.61
42	July	50	8	9.24
42	July	50	15	9.37
43	July	1	2	16.32
43	July	1	5	17.91
43	July	1	8	17.27
43	July	1	15	12.22
44	July	1	2	19.43
44	July	1	5	18.75
44	July	1	8	18.94
44	July	1	15	21.83
44	July	10	2	18.44
44	July	10	5	17.74
44	July	10	8	17.83
44	July	10	15	18.29
44	July	30	2	14.07
44	July	30	5	14.15
44	July	30	8	14.02
44	July	30	15	13.77
44	July	50	2	12.56
44	July	50	5	13.13

44	July	50	8	12.98
44	July	50	15	11.63
45	July	1	2	14.7
45	July	1	5	16.69
45	July	1	8	16.56
45	July	1	15	18.16
45	July	10	2	12.67
45	July	10	5	12.78
45	July	10	8	12.62
45	July	10	15	14.09
45	July	30	2	8.3
45	July	30	5	8.94
45	July	30	8	7.65
45	July	30	15	8.87
45	July	50	2	9.61
45	July	50	5	9.01
45	July	50	8	8.55
45	July	50	15	10.05
46	July	1	2	16.18
46	July	1	5	17.31
46	July	1	8	16.8
46	July	1	15	15.76
46	July	10	2	16.87
46	July	10	5	16.87
46	July	10	8	16.07
46	July	10	15	15.91
46	July	30	2	16.2
46	July	30	5	16.02
46	July	30	8	13.42
46	July	30	15	10.8
46	July	50	2	14.57
46	July	50	5	15.08
46	July	50	8	14.64

46	July	50	15	13.29
47	July	1	2	16.05
47	July	1	5	14.09
47	July	1	8	18.05
47	July	1	15	18.78
47	July	10	2	16.68
47	July	10	5	16.33
47	July	10	8	16.68
47	July	10	15	17.38
47	July	30	2	13.86
47	July	30	5	13.23
47	July	30	8	12.61
47	July	30	15	10.71
47	July	50	2	14.55
47	July	50	5	14.03
47	July	50	8	13.09
47	July	50	15	11.83
48	July	1	2	12.38
48	July	1	5	13.56
48	July	1	8	12.44
48	July	1	15	12.52
48	July	10	2	15.42
48	July	10	5	15.07
48	July	10	8	14.92
48	July	10	15	12.51
49	July	1	2	14.74
49	July	1	5	14.91
49	July	1	8	18.56
49	July	1	15	20.09
49	July	10	2	15.4
49	July	10	5	14.6
49	July	10	8	13.33
49	July	10	15	12.38

49	July	30	2	14.22
49	July	30	5	11.97
49	July	30	8	12.23
49	July	30	15	9.91
49	July	50	2	9.5
49	July	50	5	7.86
49	July	50	8	8.34
49	July	50	15	10.19
50	July	1	2	14.72
50	July	1	5	14.2
50	July	1	8	18.56
50	July	1	15	21.87
50	July	10	2	17.16
50	July	10	5	16.92
50	July	10	8	12.93
50	July	10	15	13.06
50	July	30	2	14.02
50	July	30	5	13.02
50	July	30	8	13.56
50	July	30	15	13.32
50	July	50	2	10.03
50	July	50	5	10.11
50	July	50	8	9.84
50	July	50	15	9.87
51	July	1	2	12.37
51	July	1	5	12.55
51	July	1	8	13.65
51	July	1	15	14.94
51	July	10	2	14.08
51	July	10	5	14.11
51	July	10	8	13.84
51	July	10	15	14.91
51	July	30	2	11.65

51	July	30	5	11.49
51	July	30	8	11.1
51	July	30	15	10.59
51	July	50	2	9.28
51	July	50	5	8.86
51	July	50	8	9.4
51	July	50	15	10.92
52	July	1	2	14.09
52	July	1	5	14.89
52	July	1	8	16.6
52	July	1	15	17.17
52	July	10	2	14.1
52	July	10	5	12.78
52	July	10	8	12.31
52	July	10	15	12.48
52	July	30	2	14.34
52	July	30	5	14.29
52	July	30	8	13.46
52	July	30	15	11.47
52	July	50	2	10.09
52	July	50	5	9.6
52	July	50	8	9.39
52	July	50	15	10.61
53	July	1	2	14.7
53	July	1	5	14.91
53	July	1	8	14.84
53	July	1	15	16.23
53	July	10	2	14.47
53	July	10	5	13.33
53	July	10	8	12.95
53	July	10	15	13.03
53	July	30	2	13.33
53	July	30	5	13.58

53	July	30	8	10.66
53	July	30	15	12.09
54	July	1	2	16.94
54	July	1	5	19.62
54	July	1	8	16.49
54	July	1	15	15.9
54	July	10	2	15.42
54	July	10	5	15.55
54	July	10	8	15.42
54	July	10	15	14.44
54	July	30	2	16.74
54	July	30	5	15.74
54	July	30	8	15.59
54	July	30	15	11.23
54	July	50	2	10.54
54	July	50	5	10.18
54	July	50	8	9.97
54	July	50	15	10.09
55	September	1	2	20.37
55	September	1	5	18.57
55	September	1	8	19.92
55	September	1	15	18.3
55	September	10	2	19.18
55	September	10	5	20.51
55	September	10	8	18.9
55	September	10	15	18
55	September	30	2	18.21
55	September	30	5	15.8
55	September	30	8	15.11
55	September	30	15	16.11
56	September	1	2	17.01
56	September	1	5	16.49
56	September	1	8	17.3

56	September	1	15	18.29
56	September	10	2	18.19
56	September	10	5	17.29
56	September	10	8	18.09
56	September	10	15	17.93
56	September	30	2	16.32
56	September	30	5	15
56	September	30	8	13.25
56	September	30	15	13.73
57	September	1	2	22.54
57	September	1	5	21.18
57	September	1	8	24.29
57	September	1	15	22.43
57	September	10	2	17.59
57	September	10	5	16.75
57	September	10	8	16.68
57	September	10	15	15.76
57	September	30	2	10.18
57	September	30	5	9.16
57	September	30	8	9.38
57	September	30	15	8.7
58	September	1	2	15.34
58	September	1	5	12.52
58	September	1	8	10.24
58	September	1	15	14.21
58	September	10	2	17.94
58	September	10	5	18.42
58	September	10	8	14.76
58	September	10	15	11.59
58	September	30	2	10.64
58	September	30	5	12.08
58	September	30	8	11.82
58	September	30	15	10.74

59	September	1	2	20.44
59	September	1	5	20.87
59	September	1	8	20.59
59	September	1	15	18.88
59	September	10	2	15.52
59	September	10	5	15.81
59	September	10	8	15.51
59	September	10	15	14.1
59	September	30	2	14.56
59	September	30	5	14.71
59	September	30	8	14.8
59	September	30	15	13.5
60	September	1	2	14.02
60	September	1	5	14.04
60	September	1	8	13.38
60	September	1	15	13.49
60	September	10	2	12.92
60	September	10	5	12.34
60	September	10	8	11.83
60	September	10	15	13.14
60	September	30	2	15.08
60	September	30	5	15.05
60	September	30	8	13.83
60	September	30	15	10.14
61	September	1	2	16.36
61	September	1	5	16.49
61	September	1	8	15.92
61	September	1	15	16.36
61	September	10	2	21.54
61	September	10	5	21.49
61	September	10	8	18.65
61	September	10	15	23.67
61	September	30	2	20.56

61	September	30	5	17.34
61	September	30	8	18.24
61	September	30	15	19.07
62	September	1	2	14.55
62	September	1	5	15.4
62	September	1	8	15.15
62	September	1	15	15.24
62	September	10	2	17.61
62	September	10	5	16.35
62	September	10	8	15.52
62	September	10	15	15.52
62	September	30	2	13.57
62	September	30	5	12.9
62	September	30	8	12.56
62	September	30	15	12.74
62	September	50	2	9.99
62	September	50	5	10.76
62	September	50	8	10.58
62	September	50	15	10.39
63	September	1	2	16.04
63	September	1	5	18.14
63	September	1	8	17.02
63	September	1	15	18.63
63	September	10	2	18.49
63	September	10	5	15.29
63	September	10	8	13.04
63	September	10	15	13.25
63	September	30	2	17.81
63	September	30	5	15.45
63	September	30	8	14.62
63	September	50	2	13.84
63	September	50	5	12.46
63	September	50	8	11.91

63	September	50	15	11.43
64	September	1	2	13.25
64	September	1	5	16.51
64	September	1	8	17.75
64	September	1	15	16.24
64	September	10	2	14.44
64	September	10	5	13.86
64	September	10	8	13.41
64	September	10	15	14.27
64	September	30	2	12.96
64	September	30	5	12.8
64	September	30	8	12.21
64	September	30	15	7.24
64	September	50	2	13.34
64	September	50	5	13.24
64	September	50	8	11.24
64	September	50	15	11.33
65	September	1	2	19.94
65	September	1	5	17.05
65	September	1	8	17.8
65	September	1	15	16.58
65	September	10	2	17.51
65	September	10	5	16.91
65	September	10	8	16.42
65	September	10	15	16.59
65	September	30	2	14.89
65	September	30	5	14.62
65	September	30	15	13.86
65	September	50	2	11.9
65	September	50	5	10.88
65	September	50	8	10.56
65	September	50	15	9.94
66	September	1	2	14.39

66	September	1	5	17.62
66	September	1	8	14.02
66	September	1	15	12.5
66	September	10	2	16.25
66	September	10	5	15.78
66	September	10	8	15.26
66	September	10	15	15.95
66	September	30	2	15.65
66	September	30	5	13.51
66	September	30	8	12.37
66	September	30	15	12.4
66	September	50	2	13.2
66	September	50	5	12.79
66	September	50	8	11.18
66	September	50	15	10.82
67	September	1	2	17.54
67	September	1	5	19.59
67	September	1	8	19.08
67	September	1	15	18.9
67	September	10	2	11.82
67	September	10	5	11.83
67	September	10	8	11.04
67	September	10	15	10.71
67	September	30	2	9.46
67	September	30	5	8.84
67	September	30	8	7.56
67	September	30	15	8.58
67	September	50	2	8.46
67	September	50	5	7.62
67	September	50	8	7.63
67	September	50	15	7.79
68	September	1	2	11.91
68	September	1	5	17.76

68	September	1	8	14.95
68	September	1	15	13.96
68	September	10	2	16.57
68	September	10	5	14.87
68	September	10	8	14.2
68	September	10	15	12.25
68	September	30	2	12.97
68	September	30	5	11.72
68	September	30	8	11.62
68	September	30	15	11.73
69	September	1	2	12.65
69	September	1	5	13.28
69	September	1	8	15.68
69	September	1	15	13.56
69	September	10	2	14.09
69	September	10	5	14.38
69	September	10	8	14.15
69	September	10	15	12.09
69	September	30	2	10.4
69	September	30	5	11.61
69	September	30	8	11.51
69	September	30	15	12.39
69	September	50	2	9.53
69	September	50	5	8.98
69	September	50	8	8.7
69	September	50	15	8.52
70	September	1	2	14.77
70	September	1	5	15.25
70	September	1	8	14.93
70	September	1	15	14.84
70	September	10	2	14.26
70	September	10	5	13.84
70	September	10	8	13.1

70	September	10	15	13.11
70	September	30	2	9.96
70	September	30	5	9.64
70	September	30	8	9.38
70	September	30	15	8.84
70	September	50	2	9.21
70	September	50	5	8.97
70	September	50	8	8.64
70	September	50	15	8.87
71	September	1	2	12.12
71	September	1	5	14.72
71	September	1	8	16.88
71	September	1	15	14.29
71	September	10	2	12.75
71	September	10	5	13.01
71	September	10	8	13.03
71	September	10	15	14.34
71	September	30	2	8.89
71	September	30	5	8.12
71	September	30	8	7.79
71	September	30	15	7.96
75	October	1	2	12.64
75	October	1	5	14.14
75	October	1	8	14.83
75	October	1	15	11.49
75	October	10	2	12.27
75	October	10	5	12.94
75	October	10	8	12.46
75	October	10	15	11.14
75	October	30	2	14.47
75	October	30	5	13.82
75	October	30	8	13.18
75	October	30	15	7.51

75	October	50	2	13.21
75	October	50	5	13.24
75	October	50	8	13.31
75	October	50	15	12.59
76	October	1	2	13.44
76	October	1	5	12.82
76	October	1	8	11.59
76	October	1	15	11.67
76	October	10	2	13.87
76	October	10	5	13.09
76	October	10	8	11.22
76	October	10	15	11.16
76	October	30	2	14.08
76	October	30	5	13.24
76	October	30	8	13.07
76	October	30	15	15.65
76	October	50	2	13.25
76	October	50	5	11.45
76	October	50	8	10.19
76	October	50	15	9.82
77	October	1	2	16.55
77	October	1	5	14.3
77	October	1	8	15.97
77	October	1	15	12.4
77	October	10	2	11.89
77	October	10	5	10.66
77	October	10	8	10.71
77	October	10	15	7.37
77	October	30	2	13.65
77	October	30	5	12.9
77	October	30	8	12.53
77	October	30	15	11.17
77	October	50	2	13.71

77	October	50	5	13.4
77	October	50	8	13.52
77	October	50	15	14.07
78	October	1	2	18.6
78	October	1	5	18.48
78	October	1	8	17.29
78	October	1	15	15.54
78	October	10	2	17.08
78	October	10	5	16.49
78	October	10	8	15.77
78	October	10	15	16.36
78	October	30	2	13.59
78	October	30	5	13.61
78	October	30	8	13.32
78	October	30	15	18.12
78	October	50	2	9
78	October	50	5	7.65
78	October	50	8	8.56
78	October	50	15	12.07
79	October	1	2	14.47
79	October	1	5	10.26
79	October	1	8	12.43
79	October	1	15	16.92
79	October	10	2	17.01
79	October	10	5	15.57
79	October	10	8	15.34
79	October	10	15	17.52
79	October	30	2	16.13
79	October	30	5	15.71
79	October	30	8	14.8
79	October	30	15	9.75
79	October	50	2	11.52
79	October	50	5	10.81

79	October	50	8	10.03
79	October	50	15	11.41
80	October	1	2	17.86
80	October	1	5	15.94
80	October	1	8	15.29
80	October	1	15	16.52
80	October	10	2	17.53
80	October	10	5	16.07
80	October	10	8	16.32
80	October	10	15	15.3
80	October	30	2	19.13
80	October	30	5	18.27
80	October	30	8	16.62
80	October	30	15	14.72
80	October	50	2	13.76
80	October	50	5	12.95
80	October	50	8	13.22
80	October	50	15	15.31
81	October	1	2	18.14
81	October	1	5	15.12
81	October	1	8	16.7
81	October	1	15	19.94
81	October	10	2	19.17
81	October	10	5	16.79
81	October	10	8	17.59
81	October	10	15	20.25
81	October	30	2	15.39
81	October	30	5	14.36
81	October	30	8	16.68
81	October	30	15	14.12
81	October	50	2	14.49
81	October	50	5	14.04
81	October	50	8	12.97

81	October	50	15	17.13
82	October	1	2	23.22
82	October	1	5	21.34
82	October	1	8	20.4
82	October	1	15	22.39
82	October	10	2	14.33
82	October	10	5	13.45
82	October	10	8	15.56
82	October	10	15	17.45
82	October	30	2	13.54
82	October	30	5	12.55
82	October	30	8	12.42
82	October	30	15	13.89
82	October	50	2	19.02
82	October	50	5	11.5
82	October	50	8	10.71
82	October	50	15	13.46
83	October	1	2	20.4
83	October	1	5	18.91
83	October	1	8	20.88
83	October	1	15	20.25
83	October	10	2	13.35
83	October	10	5	11.9
83	October	10	8	12.4
83	October	10	15	14.69
83	October	30	2	10.12
83	October	30	5	10.47
83	October	30	8	10.62
83	October	30	15	12.94
83	October	50	2	14.18
83	October	50	5	11.78
83	October	50	8	9.91
83	October	50	15	10.81

84	October	1	2	21.98
84	October	1	5	23.6
84	October	1	8	21.99
84	October	1	15	22.09
84	October	10	2	16.35
84	October	10	5	16.02
84	October	10	8	16.75
84	October	10	15	18.63
84	October	30	2	20.23
84	October	30	5	17.5
84	October	30	8	17.06
84	October	30	15	17.93
84	October	50	2	11.09
84	October	50	5	10.39
84	October	50	8	8.67
84	October	50	15	13.62
85	October	1	2	21.45
85	October	1	5	24.01
85	October	1	8	24.59
85	October	1	15	21.96
85	October	10	2	21.47
85	October	10	5	20.95
85	October	10	8	22.4
85	October	10	15	20.98
85	October	30	2	18.54
85	October	30	5	17.08
85	October	30	8	17.26
85	October	30	15	16.74
85	October	50	2	14.24
85	October	50	5	13.65
85	October	50	8	13.62
85	October	50	15	15.36
86	October	1	2	18.84

86	October	1	5	17.83
86	October	1	8	20.79
86	October	1	15	21.37
86	October	10	2	20.84
86	October	10	5	20.86
86	October	10	8	20.61
86	October	10	15	20.6
86	October	30	2	13.74
86	October	30	5	13.81
86	October	30	8	13.67
86	October	30	15	14.82
86	October	50	2	13.59
86	October	50	5	10.64
86	October	50	8	10.24
86	October	50	15	9.8

Data set of rH values in plain sediment (control)

Core	Time	Sediment depth (cm)	rH
1	April	30	11.0609
1	April	10	14.4181
1	April	1	14.2347
1	April	50	8.72855
2	April	50	9.38541
2	April	1	15.377
2	April	10	10.244
2	April	30	8.94383
3	April	30	8.29761
3	April	1	15.903
3	April	50	8.03633
3	April	10	11.7651
4	April	50	6.86696
4	April	1	12.6244
4	April	10	7.98171
4	April	30	7.03375
5	April	50	8.17289
5	April	1	19.3456
5	April	10	16.9128
5	April	30	13.9586
6	April	50	6.92554
6	April	10	18.5797
6	April	30	9.14307
6	April	1	19.1341
7	April	50	15.1893
7	April	30	16.1562
7	April	10	14.5757
7	April	1	16.3548
8	April	1	22.313
8	April	50	8.76967
8	April	30	12.809
8	April	10	23.4402
9	April	50	7.47597
9	April	1	15.4164
9	April	10	14.0427
9	April	30	7.61729
10	April	1	19.0607
10	April	50	6.71663
10	April	30	8.15151
10	April	10	14.9915
11	April	50	7.28664
11	April	1	16.2322
11	April	30	7.95815
11	April	10	14.5816
12	April	1	17.1854
12	April	10	17.2324
12	April	50	7.22016

12	April	30	10.7003
13	July	1	13.6826
13	July	50	7.05174
13	July	30	7.37857
13	July	10	10.974
14	July	10	17.1454
14	July	50	7.0918
14	July	1	19.0541
14	July	30	7.39881
15	July	50	7.67887
15	July	1	13.4896
15	July	10	14.7233
15	July	30	8.09923
16	July	50	7.16247
16	July	30	7.48979
16	July	1	20.8174
16	July	10	12.9926
17	July	50	8.0345
17	July	30	7.73215
17	July	1	18.0535
17	July	10	16.2479
18	July	1	16.0294
18	July	10	14.0242
18	July	30	8.20064
18	July	50	7.4775
19	July	30	16.9313
19	July	50	11.7612
19	July	10	20.0863
19	July	1	18.6905
20	July	50	7.89567
20	July	30	11.7154
20	July	10	12.3612
20	July	1	10.6154
21	July	50	7.44209
21	July	1	15.8221
21	July	10	16.3669
21	July	30	8.37579
22	July	30	8.15288
22	July	10	14.0923
22	July	1	17.0669
22	July	50	7.06561
23	July	10	18.2017
23	July	30	18.7658
23	July	50	12.7043
23	July	1	18.8021
24	July	1	17.4269
24	July	10	16.508
24	July	30	9.64118
24	July	50	8.30757
25	September	10	8.306

25	September	50	7.20522
25	September	30	7.23863
25	September	1	12.7824
26	September	30	7.77228
26	September	1	16.0311
26	September	10	15.1571
26	September	50	7.31875
27	September	30	9.64678
27	September	1	16.4381
27	September	10	17.1314
27	September	50	7.65881
28	September	10	14.9102
28	September	50	7.58552
28	September	1	19.8612
28	September	30	9.85331
29	September	10	21.688
29	September	30	10.1606
29	September	50	8.44606
29	September	1	19.8074
30	September	1	16.8651
30	September	30	7.37204
30	September	10	17.3452
30	September	50	7.10516
31	September	1	12.8838
31	September	10	10.0762
31	September	30	8.01829
31	September	50	7.6975
32	September	10	18.02
32	September	30	14.4781
32	September	50	8.07215
32	September	1	24.3585
33	September	50	8.17293
33	September	30	13.7634
33	September	10	16.1587
33	September	1	12.0263
34	September	10	21.5046
34	September	30	7.54924
34	September	50	7.48706
34	September	1	22.516
35	September	50	7.56427
35	September	30	10.2532
35	September	10	22.3608
35	September	1	21.1881
36	September	50	7.20851
36	September	1	21.8051
36	September	10	14.8154
36	September	30	8.44294
37	October	1	23.8509
37	October	50	12.7621
37	October	30	17.4177

37	October	10	23.3012
38	October	50	13.6864
38	October	30	14.2095
38	October	10	22.2624
38	October	1	22.8703
39	October	50	7.8707
39	October	10	16.6969
39	October	1	16.2712
39	October	30	8.42148
40	October	1	19.6369
40	October	10	8.85167
40	October	30	7.99848
40	October	50	6.99624
41	October	1	18.0488
41	October	30	10.8635
41	October	10	18.6432
41	October	50	7.6579
42	October	50	7.8046
42	October	10	17.6223
42	October	1	22.9308
42	October	30	8.78093
43	October	1	21.9463
43	October	10	16.3868
43	October	30	9.73543
43	October	50	11.741
44	October	10	17.4864
44	October	50	7.64891
44	October	30	9.06724
44	October	1	21.5729
45	October	1	21.9161
45	October	50	7.31579
45	October	30	14.8871
45	October	10	15.8117
46	October	50	7.73108
46	October	1	19.7502
46	October	10	22.0127
46	October	30	11.7376
47	October	50	7.53591
47	October	1	17.9644
47	October	10	18.0154
47	October	30	10.5647
48	October	30	9.9638
48	October	10	17.3994
48	October	1	16.3674
48	October	50	7.10899
